# Effect of Combining Surfactants with Potato Protein Hydrolysates on Their Emulsifying and Antioxidant Properties in Fish-Oil-in-Water Emulsions

**DOI:** 10.3390/foods14111974

**Published:** 2025-06-02

**Authors:** Cansu Yay, Betül Yesiltas, Charlotte Jacobsen

**Affiliations:** 1 Institute of Biotechnology, Gebze Technical University, 41400 Gebze, Kocaeli, Türkiye; cansuyay@gtu.edu.tr; 2 National Food Institute, Technical University of Denmark, 2800 Kgs. Lyngby, Denmark; chja@food.dtu.dk

**Keywords:** potato peptides, emulsifier, lipid oxidation, antioxidant, low-fat emulsions, Tween 20, DATEM

## Abstract

This study investigated the emulsifying and antioxidant properties of potato protein hydrolysates (PPHs) obtained through enzymatic hydrolysis with trypsin, aiming to utilize them as natural emulsifiers in 5 wt% fish-oil-in-water emulsions. Unfractionated and fractionated PPH fractions (>10 kDa, 5–10 kDa, 0.8–5 kDa, and <0.8 kDa) in combination with surfactants (Tween 20 or DATEM) were evaluated. Unfractionated PPH alone resulted in unstable emulsions; however, combining it with 67 wt% DATEM or Tween 20 improved physical stability. Smaller PPH fractions (<10 kDa) produced smaller droplet sizes (0.352–0.764 μm) with DATEM, whereas for Tween 20-stabilized emulsions, the smallest droplet size was observed with unfractionated PPH (1.051 ± 0.015 µm). Notably, the 5–10 kDa fraction exhibited the best oxidative stability when combined with Tween 20, likely due to its antioxidant properties. While further refinement is necessary to improve PPHs’ effectiveness as standalone emulsifiers, their potential is evident.

## 1. Introduction

Emulsions are thermodynamically unstable systems that require emulsifiers to provide physical stability. Emulsifiers function by reducing the interfacial tension between oil and water, thereby keeping emulsions physically stable for a period of time. Oil-in-water emulsions are a widely used type of emulsion in the food industry, where both physical and oxidative stability are crucial factors. In particular, omega-3 polyunsaturated fatty acids (PUFAs) offer several health benefits, including the reduced prevalence of cardiovascular disease and inflammation and improved cognitive functions and brain development [1,2,3]. However, the high degree of unsaturation in fish oil makes it susceptible to oxidation, which ultimately reduces its nutritional and organoleptic properties [4,5]. Thus, maintaining the oxidative stability of PUFA-rich oils remains a challenge in food formulation, despite the aforementioned health benefits.

There are many factors to consider in the design of such emulsion systems in the prevailing molecular environment. These include interfacial properties such as charge, position; the partition of surfactant, antioxidant molecules, and transition metals if present; and the number of adsorbed and free surfactant molecules [6,7]. Therefore, creating an appropriate molecular environment that promotes physical and oxidative stability is essential, with emulsifiers playing a central role. Surfactants are generally preferred as emulsifiers because their amphiphilic structure, containing both hydrophobic and hydrophilic regions, allows them to reduce interfacial tension during emulsification and enhance physical stability. Additionally, emulsifiers with antioxidant properties can further enhance oxidative stability, which can be accomplished in a variety of ways: sequestering or deactivating the transition metals, scavenging free radicals, or providing a physical barrier [8].

Sustainable food consumption and the demand for all-natural foods have focused the attention of the food industry on the valorization of side streams and the replacement of widely used synthetic ingredients with natural ones. The role of protein hydrolysates increases in the food industry in this regard, and they can be obtained in various ways such as by enzymatic or chemical hydrolysis or by microbial fermentation of different protein sources [9,10]. The properties of protein hydrolysates are largely determined by the source of the protein, the hydrolysis technique and conditions, and the fractionation and purification processes utilized [11]. The potato starch industry produces ~240,000 tons of potato protein per year, making it a good, abundant, non-animal source that can contain a variety of embedded properties [12]. Emulsifying activity can be one of these embedded properties. A novel bioinformatics and proteomics-guided approach has been used to predict and obtain emulsifier peptides from potato side streams, and in a follow-up study, these peptides were used as emulsifiers and their emulsifying properties were evaluated [13,14]. On the basis of the same proteomics and bioinformatic approach, a targeted hydrolysis workflow for releasing abundant emulsifier peptides from a potato protein source has been demonstrated in a previous study [15]. This approach is a novel, cost- and time-efficient way of releasing functional peptides. In addition to that, potato proteins are associated with foaming and antioxidant properties [16,17]. Thus, despite its low protein content, the abundance of this crop makes it a good candidate for the valorization of the side-stream of the potato starch processing industry [12].

In a previous study, targeted trypsin hydrolysis followed by ultrafiltration was used to produce potato protein hydrolysate (PPH) fractions [18]. The resulting PPH fractions were used as a dual-functional ingredient with both emulsifying and antioxidant properties. It was used as the sole emulsifying agent in 5 wt% oil-in-water emulsions, and PPHs with a molecular weight higher than 5 kDa (5–10 kDa and >10 kDa) provided better physical and oxidative stability during storage than smaller fractions (<0.8 kDa and 0.8–5 kDa) and unfractionated PPH. Furthermore, these PPH fractions (5–10 kDa and >10 kDa) were also promising for radical scavenging and metal chelating activities. While unfractionated PPHs and fractionated PPHs displayed some potential for contributing to oxidative stability as the only emulsifiers in the system, likely due to their metal-chelating and radical scavenging activities, they were not sufficient to fully prevent the increase in peroxide value (PV), the loss of tocopherols, and the formation of secondary oxidation products. Therefore, they were not effective as sole emulsifiers. Combining PPHs with surfactants could improve physical stability, while utilizing the antioxidant properties of PPHs can help mitigate lipid oxidation.

The objective of this study was to determine whether a combination of potato-juice-derived PPHs (with antioxidant and emulsifying capabilities) in the fractions >10 kDa, 5–10 kDa, 0.8–5 kDa, and <8 kDa and an unfractionated hydrolysate, along with the commonly used commercial surfactants diacetyl tartaric acid ester of mono- and diglycerides (DATEM) and Tween 20 (TW20), could improve the physical and oxidative stability of 5 wt% fish-oil-in-water emulsions. Tween 20 and DATEM were selected for this study due to their physiochemical properties and widespread use in food emulsions. Tween 20 is a nonionic surfactant and stabilizes emulsions predominantly via steric repulsion [19,20]. DATEM is a low-molecular-weight anionic emulsifier and is commonly used in baking [7,21]. The inclusion of these two surfactants in combination with PPHs allowed us to explore different stabilization mechanisms. First, small-scale emulsions (a total of 2 g) were prepared to determine the optimum surfactant-to-protein hydrolysate ratio to obtain physical stability. Then, according to the results of the first stage, large-scale emulsions (250 g) were produced to investigate both the physical and oxidative stability of these emulsions.

## 2. Materials and Methods

### 2.1. Materials

Fish oil (cod liver oil) was obtained from Vesteraalens A/S (Sortland, Norway). The peroxide value of the fish oil was 0.31 ± 0.01 meq O_2_/kg oil. Fatty acid methyl ester (FAME) analysis by GC-FID was used to determine the fatty acid composition of the fish oil in a previous study and reported as the following. C14: 0 (3.7% of total fatty acids); C16: 0 (9.0%); C16: 1n-7 (9.6%); C18: 0 (1.9%); C18: 1n-9 (15.8%); C18: 1n-7 (4.5%); C18: 2n-6 (2.4%); C18: 3n-3 (1.0%); C20: 1n-9 (13.7%); C20: 5n-3 (8.4%); C22: 1n-11 (5.6%); and C22: 6n-3 (10.7%) [18]. The tocopherol content of the fish oil was measured as α-tocopherol, 176 ± 5.2 µg/g oil; γ-tocopherol, 109 ± 3.2 µg/g oil; δ-tocopherol, and 39 ± 1.1 µg/g oil, according to the AOCS Official Method [22]. PV of DATEM was 11.58 ± 1.81 meq O_2_/kg oil. All chemicals and solvents used were of analytical grade.

The unfractionated and size-fractionated PPHs were prepared according to the method described by Yesiltas, García-Moreno et al. [18]. Briefly, PPH was produced by targeted Trypsin hydrolysis (Pancreatic Trypsin Novo, 6.0 S, 6.0 AU/g) (Novonesis A/S (former Novozymes A/S), Bagsværd, Denmark) (0.05% *w*/*v* Trypsin at pH 8, 50 °C for 24 h), where Trypsin was specifically selected based on a proteomics and bioinformatics approach to release peptides with emulsifying properties. Among the tested proteases (Neutrase, Alcalase, Flavourzyme, and Trypsin), Trypsin was identified as the most effective, as predicted by in silico analysis and confirmed through in vitro experiments [15]. The hydrolysate was then fractionated using sequential ultrafiltration with different molecular weight cut-off membranes (10 kDa, 5 kDa, and 0.8 kDa). The protein content of the unfractionated and fractionated potato protein hydrolysates was measured using the Dumas principle and is listed in Table 1.

### 2.2. Production of Small-Scale Emulsions

To determine the optimum surfactant/protein hydrolysate ratio in the emulsion, a total of 2 g of oil-in-water emulsions (5 wt%) were prepared with varying percentages of surfactants (DATEM and TW20) (100%, 67%, 50%, and 33 wt%) and the unfractionated PPH. The oil phase ratio, 5% (*w*/*w*), was selected based on previous studies that utilized similar concentrations for screening emulsion stability and antioxidant performance in protein hydrolysate stabilized systems [18,23]. The total content of surfactants and PPH was 0.2 wt% in the emulsion. The aqueous phase was made by dissolving the unfractionated PPH in 10 mM sodium acetate–10 mM imidazole buffer (pH 7) and stirring at 4 °C overnight. The next day, DATEM and TW20 surfactants were added into the aqueous phase followed by the addition of rapeseed oil, and homogenization was started. Rapeseed oil was used in the preparation of small-scale emulsions for the initial screening of physical stability. Since oxidative stability was not evaluated at this stage, rapeseed oil served as a suitable oil phase. Primary homogenization was performed by mixing at 18,000 rpm for 30 s by using a POLYTRON^®^ PT1200E (Kinematic Inc., New York, NY, USA). For secondary homogenization, a sonicator Microson XL2000 equipped with a P1 probe (Misonix, Inc., New York, NY, USA) was used. Emulsions were homogenized at an amplitude of 75% (maximum amplitude of 180 µm), running two passes of 30 s with a break of 1 min between passes to prevent excessive temperature increase during sonication. The emulsions were surrounded by iced water to minimize the increase in temperature throughout the sonication. Emulsions were prepared in duplicate and stored for 7 days at 25 °C in darkness and sampling was performed to evaluate the physical stability with the measurement of droplet size and zeta potential.

It is important to note that different oils were used in the two experimental stages: rapeseed oil in the small-scale screening and fish oil in the large-scale trials. Rapeseed oil, rich in monounsaturated fatty acids, is relatively stable against oxidation, while fish oil contains high levels of polyunsaturated fatty acids, which are highly prone to oxidation [24,25]. Thus, rapeseed oil was selected for initial screening purposes, while fish oil was selected to assess oxidative stability under more realistic and sensitive conditions.

### 2.3. Production of Large-Scale Emulsions and Storage Experiment

Large-scale emulsions were produced for all fractions and unfractionated PPHs. A 5 wt% fish-oil-in-water emulsion (250 g) was stabilized with 0.2 wt% of surfactant and protein hydrolysate in total, and the percentages of surfactant and protein hydrolysates were 67% and 33%, respectively, and determined based on the physical stability observed in the small-scale emulsions. Since the protein content of each hydrolysate was different, the required amount to provide 33 wt% was calculated for each hydrolysate. Firstly, PPHs were dissolved in 10 mM sodium acetate–10 mM imidazole buffer (pH 7). They were kept stirring in the refrigerator in darkness overnight to make sure they dissolved completely. The next day, TW20 and DATEM surfactants were added to the solutions. Pre-homogenization was performed using ultraturrax (Ystral, Ballrechten-Dottingen, Germany) for 3 min at 16,000 rpm. Fish oil was added to the aqueous phase during the first minute of pre-homogenization. Secondary homogenization was performed using a Microfluidizer (M110L Microfluidics, Newton, MA, USA) equipped with a ceramic interaction chamber (CIXC, F20Y, internal dimension 75 μm) at 9 kpsi and three passes. Two extra emulsions that only contained TW20 and DATEM (T100% and D100%), respectively, were prepared and used as controls. After the homogenization was completed, 50 µM FeSO_4_ solution and sodium azide (0.05%) were added as a prooxidant and antimicrobial solution, respectively, to the final emulsions, which were then stirred with a spoon. The final pH of the emulsions was measured and ranged from 6.40 to 6.75 for the emulsions prepared with DATEM and 6.90 to 6.98 for the emulsions prepared with TW20. A 1M NaOH solution was added to increase the pH of the emulsions prepared with DATEM, and the final pH of these emulsions was 7.00 ± 0.02. The emulsions were stored for 9 days at 25 °C in darkness. Samples were collected for physical characterization and oxidative stability analyses during storage and were stored at −40 °C until analysis.

### 2.4. Physical Stability of Emulsions

While classical measurements like emulsifying activity and stability index were not performed, physical stability was assessed via the Turbiscan stability index, droplet size, and zeta potential, which provide insights into emulsifying performance under realistic application conditions.

#### 2.4.1. Emulsion Stability

Turbiscan Tower (Formulaction-Microtrac, Toulouse, France) was used to measure the physical stability of the large-scale, low-fat emulsions for 18 h on the production day (day 0) of the TW20 emulsions and on the first day of the DATEM emulsions. Ten milliliters of each emulsion was transferred into special vials and placed into the instrument and scanned measuring backscattering (BS) and transmission (T). Turbiscan stability index (TSI) is reported at the end of the process by the instrument and is calculated based on T and BS values in the following equation:

(1) $$TSI\left(t\right)=\frac{1}{N_{h}}\sum_{t_{i}}^{t_{max}}\sum_{z_{i}=z_{min}}^{z_{max}}\left|BST\left(t_{i},z_{i}\right)-BST(t_{i-1},z_{i})\right|$$

where t_max_ is the measurement point at time t, when the TSI is calculated; z_min_ and z_max_ are the lower and upper height limit, respectively; N (h) = (z_max_ − z_min_)/Δh is the number of height positions for the scan; and BST is the signal that is considered (BS when T < 0.2%, otherwise T is taken) (Formulaction, 2021). The stability of the emulsions was evaluated on the day of production for emulsions prepared using TW20 and on the first day for emulsions prepared using DATEM, as only 6 samples can be run at the same time in the Turbiscan Tower. Measurements were run as a single determination for checking if the stability of the emulsions was acceptable.

#### 2.4.2. Droplet Size Distribution

The droplet size distribution of the emulsions was measured by the laser diffraction technique using Mastersizer 2000 (Malvern Instruments, Ltd., Worcestershire, UK) on days 1 and 7 for small-scale emulsions and on days 1 and 9 for large-scale emulsions. Emulsions were diluted in recirculating water (3000 rpm) until they reached an obscuration of 12–15%. The refractive indices of sunflower oil (1.469) and water (1.330) were used as particle and dispersant, respectively. Measurements were carried out in duplicate (or triplicate if necessary). Results were given as the surface-weighted (D [3,2]) and volume-weighted (D [4,3]) mean diameters.

#### 2.4.3. Zeta Potential

The zeta potential of the emulsions was measured on day 1 using Zetasizer Nano ZS (Malvern instruments Ltd., Worcestershire, UK). Prior to the analysis, 80 µL of each emulsion was diluted in 40 mL of 10 mM sodium acetate–10 mM imidazole buffer (pH 7) and vortexed. Then, samples were transferred to the DTS-1070 disposable folded capillary cell (Malvern Instruments, Ltd., UK). The zeta potential range was set to −100 to +50 mV, and samples were analyzed with 100 runs at 25 °C. Measurements from the same sample were carried out in duplicate (or triplicate when necessary).

### 2.5. Oxidative Stability of Emulsions

Samples were collected on days 0, 1, 2, 5, and 9 during the storage experiment and oxidative stability analyses were performed for those samples.

#### 2.5.1. Peroxide Value

Prior to peroxide value (PV) determination, lipids in the emulsions were extracted based on the method described by Bligh and Dyer [26] with a modification to reduce the amount of solvent used chloroform/methanol (1:1, *v*/*v*). Two extractions were made from each emulsion sample. The colorimetric ferric thiocyanate method described by Shantha and Decker [27] was used to determine PV. The measurements were performed at 500 nm (Shimadzu UV-1280, Holm & Halby, Brøndby, Denmark) and were made in duplicate.

#### 2.5.2. Tocopherol Content

The AOCS Official Method Ce 8-89 was followed to monitor changes in tocopherols [22]. Approximately 2 g of the lipid extract from the Bligh and Dyer extraction was weighed and evaporated under nitrogen and then redissolved in 1 mL n-heptane, and these solutions were transferred to separate vials. An Agilent 1100 series HPLC (Agilent Technologies, Palo Alto, CA, USA) equipped with a fluorescent detector was used. The characteristics of the HPLC system are as follows: mobile phase of heptane/2-propanol (100/0.4, *v*/*v*), isocratic pump with a flow rate of 1.0 mL/min, injection volume 20 μL, Waters Spherisorb 3 μm Silica column (4.6 mm I.D. × 150 mm), Waters Spherisorb 5 μm Silica guard column (4.6 mm I.D. × 10 mm), and fluorescence detection performed at 290 nm (excitation wavelength) and 330 nm (emission wavelength). Tocopherol standard mix including α, β, γ, and δ-tocopherol standards was used to quantify the tocopherols in the emulsions. Measurements were performed in duplicate and expressed in μg of each tocopherol/g of oil.

#### 2.5.3. Secondary Volatile Oxidation Compounds—Dynamic Headspace Coupled with GC-MS

Volatile compounds were measured using dynamic headspace combined with GC-MS. Approximately 4 g of the emulsion was added to a pear-shaped bottle with 5 mL distilled water and 30 mg of the internal standard (4-methyl-1-pentanol), and to extract the volatiles, a pear-shaped bottle was subjected to a water bath at 45 °C for 30 min under a nitrogen flow of 150 mL/min and volatiles trapped in Tenax GR tubes. Before putting tubes into an Automatic Thermal Desorber (ATD-400, Perkin Elmer, Norwalk, CT, USA), which was connected to a GC (GC Agilent 6890 N, Palo Alto, CA, USA), tubes were flushed with nitrogen (50 mL/min) for 20 min. The GC system had the following characteristics: DB 1701 fused silica capillary column (0.25 mm I.D. × 30 m, 1μm film thickness; J&W Scientific, Folsom, CA, USA). The oven conditions for the 5% fish-oil-in-water emulsions were described previously by Yesiltas et al. [18]. The initial temperature of the oven was 45 °C for 5 min, increasing by 1.5 °C/min until reaching 55 °C, increasing by 2.5 °C/min until reaching 90 °C, and increasing by 12 °C/min until 220 °C and kept for 4 min. The volatile compounds were separated by MS (MS Agilent 5973, Agilent Technologies, USA) at the electron ionization mode of 70 eV. Mass-to-charge ratio scans were performed between 30 and 250. MS library searches (Wiley 138 K, John Wiley, and Sonsi Hewlett–Packard) were carried out to identify volatile compounds. Quantification was performed for the following volatile compounds: 1-penten-3-ol, 1-penten-3-one, hexanal, *(E*,*E)*-2,4-heptadienal, 2-ethyl furan, 2,3-pentanedione, pentanal, 1-pentanol, *(E)*-2-hexenal, heptanal, *(Z)-*4-heptanal, *(E)-*2-heptanal, octanal, *(E*,*E)*-2,4-deceadienal, *(E)*-2-butenal, and 1-octen-3-ol. To quantify volatile compounds in the emulsions, a stock standard solution containing these volatile compounds was used to prepare the calibration curve, and seven different concentrations of diluted stock solution were added to a fresh DATEM and TW20 containing emulsion, respectively. Separate calibration curves, one for the DATEM-containing emulsion and one for the TW20-containing emulsion, were made to investigate whether the matrix, the presence of DATEM or TW20, had an effect on the formation or release of the volatiles. It was then observed that these two calibration curves were similar Therefore, the calibration curve of the DATEM-containing emulsion was used for the calculations. The analysis for volatiles was performed in triplicate, and the results are expressed as ng/g sample.

### 2.6. Statistical Analysis

Data were analyzed using Statgraphics Centurion 18 software (Statistical Graphics Corp., Rockville, MD, USA). One-way analysis of variance (ANOVA) was followed by multiple sample comparison analysis using Tukey’s post hoc test at a 95% confidence level (differences were considered significant at *p* < 0.05).

## 3. Results and Discussion

### 3.1. Determination of Optimal Surfactant to Protein Ratio in Small-Scale Emulsion Study

A total of 0.2% by weight of the total emulsion was chosen as the total protein hydrolysate and surfactant content in the emulsion. The results of the determination of the droplet size distributions obtained with oil-in-water emulsions containing different ratios of surfactant-to-unfractionated-potato-protein-hydrolysate (PPH) ratios are shown in Table 2. The highest volume-weighted mean was 5.30 ± 0.56 µm on the emulsion production day, belonging to the emulsion containing only unfractionated PPH; this value increased to 18.936 ± 2.503 µm, indicating instability, at the end of the storage period. Therefore, the use of PPH as the sole emulsifier component in the system was not an option. In a previous study conducted with PPHs produced by Alcalase treatment, PPHs alone were also not sufficient to exhibit emulsifying properties, resulting in aggregation and creaming; however, the use of TW20 as a co-emulsifier provided stability in these emulsions [28].

The volume and surface-weighted mean of the DATEM emulsions were lower than those of the TW20 emulsions on day 1 after production (Table 2). However, the DATEM emulsions showed an increase in droplet size at the end of storage, whereas this was not the case for TW20 emulsions. Emulsions prepared with TW20 had droplet sizes between 0.79–2.77 µm in terms of volume weighted mean diameter and 0.302–0.412 µm in terms of surface-weighted mean. For zeta potential, the emulsion containing unfractionated PPH had a value of −56.13 ± 1.80 mV, which is similar to the zeta potential obtained by Yesiltas et al. [18] (−59.6 ± 1.3 mV) using unfractionated PPH alone in a low-fat emulsion. Emulsions prepared with DATEM had more negative zeta potential than those prepared with TW20, as expected, due to low electrostatic repulsion between oil droplets because of TW20’s nonionic nature. Adding PPH to DATEM emulsions increased their zeta potential, whereas the opposite was the case for TW20 emulsions.

In terms of both mean droplet size and zeta potential, 67 wt% surfactant and 33 wt% PPH provided good stability for both types of surfactants, and this ratio was therefore selected for large-scale emulsions for all PPH fractions and unfractionated PPH with the aim of evaluating their effect on physical and oxidative stability.

### 3.2. Physical Stability of the Emulsions Produced on a Large Scale with Fractions of PPH

The 5% *w*/*w* fish-oil-in-water emulsions produced on a large scale (250 g) stabilized with the combinations of 33% *w*/*w* fractionated (<0.8 kDa to >10 kDa) or unfractionated potato protein hydrolysates combined with 67% *w*/*w* surfactant (DATEM or TW20) resulted in physically stable emulsions right after homogenization, as no creaming or aggregation was observed (Figure S1). The physical stability of the emulsions was investigated in terms of the Turbiscan stability index, changes in droplet size during storage, and zeta potential on the day after production.

It is important to note that while the primary homogenization method, ultra-turrax, was the same across both experimental phases, the secondary homogenization techniques differed: ultrasonication was used in the small-scale screening phase, whereas high-pressure microfluidization was applied in the large-scale experiments. This difference may have contributed to variations in physical stability, as high-pressure homogenization generally produces finer and more uniform emulsions compared to sonication [29]. These differences reflect the practical objective of each phase, rapid screening of several formulations versus validation under conditions more relevant to industrial application. This should therefore be considered when comparing data from different stages, as homogenization methods can significantly affect emulsion structure.

#### 3.2.1. Emulsion Stability-TSI

**Figure 1:**
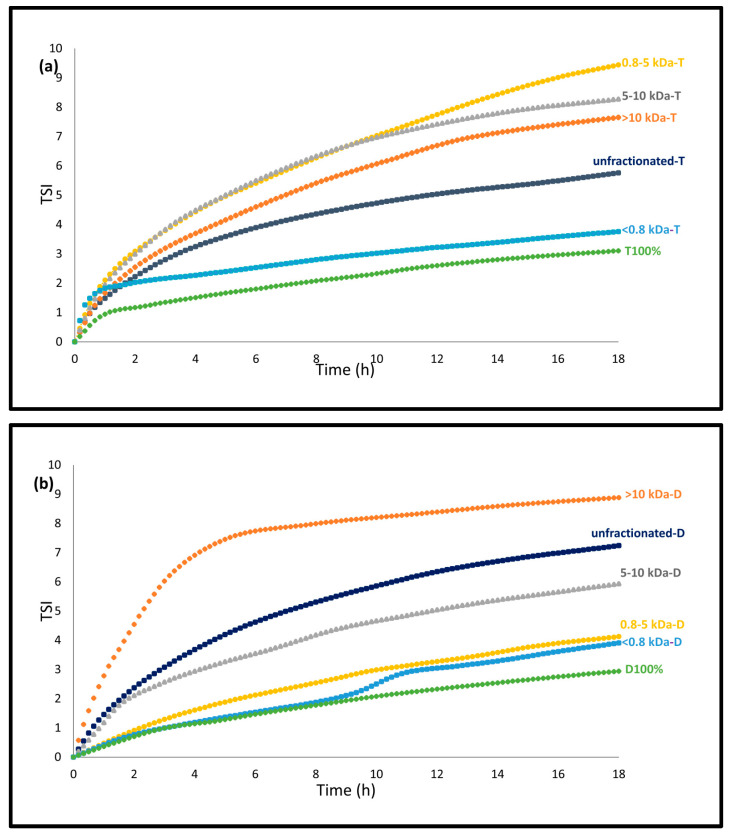
Emulsion stability of the 5 wt% fish-oil-in-water emulsions produced on a large scale (250 g) stabilized with the combinations of 33% *w*/*w* fractionated (<0.8 kDa to >10 kDa) or unfractionated potato protein hydrolysates with (**a**) 67% *w*/*w* TW20 containing emulsions measured on day 0, and (**b**) 67% *w*/*w* DATEM containing emulsions measured on day 1. Both measurements were conducted for 18 h.

Emulsion stability was assessed on the day of production for emulsions containing TW20 and on the first day for emulsions containing DATEM due to the Turbiscan Tower’s capacity limit of six samples per run. The results of the Turbiscan stability index (TSI) calculations are shown in Figure 1, where TSI values below 3 are considered physically stable emulsions (Formulaction, 2021). Among the TW20 emulsions, T100% showed the highest stability, with a TSI of 3.16 at the end of the measurement, while the other TW20 emulsions exhibited higher TSI values. The emulsion containing the <0.8 kDa fraction and TW20 had a TSI that was most similar to that of T100%, with a final TSI of 3.76. In contrast, the 0.8–5 kDa-T emulsion showed the lowest stability, reaching a TSI of 9.44 (Figure 1a). Similarly, for the DATEM emulsions, D100% demonstrated the greatest stability, with a TSI value of 2.94 at the end of analysis (Figure 1b). This was followed by the 0.8 kDa-D and 0.8–5 kDa-D emulsions, which had TSI values of 3.91 and 4.13, respectively. Conversely, the emulsion containing the >10 kDa fraction showed the highest TSI (8.89), indicating the lowest physical stability among the DATEM emulsions.

#### 3.2.2. Droplet Size Distribution and Zeta Potential

The droplet size distributions of emulsions prepared with 5 wt% fish-oil-in-water emulsion in 0.2 wt% of surfactants (DATEM, TW20, respectively) and fractions of potato protein hydrolysates are shown in Table 3, Figure S2, and Table S1 (Supplementary Materials). For both surfactants, the smallest droplet sizes were observed in the emulsions prepared with the surfactant alone, i.e., the D100% and T100% emulsions. These emulsions also exhibited monomodal distributions throughout their storage (Figure S2). Among the DATEM emulsions, combinations with low-molecular-weight PPH fractions (<0.8 kDa, 0.8–5 kDa) also showed monomodal distributions. On the other hand, fractions >5 kDa and unfractionated hydrolysate showed bimodal distributions. For TW20 emulsions, all combinations with PPH fractions and unfractionated hydrolysate resulted in bimodal distributions. The highest volume-weighted mean droplet size was observed in the emulsion containing DATEM and the >10 kDa fraction, with a value of 2.43 ± 0.04 µm on the first day and 2.51 ± 0.00 µm by the end of storage. This was also in agreement with the high TSI value, supporting the observed physical instability. Interestingly, decreasing the molecular weight of the PPH fraction in DATEM emulsions led to smaller droplet sizes, with the <0.8 kDa fraction producing the smallest and most stable droplets among all DATEM–PPH combinations. On the other hand, for TW20 emulsions, T100% exhibited the smallest droplet size (D [4,3] = 0.32 ± 0.00 µm), followed by the unfractionated-T (1.05 ± 0.02 µm). Emulsions containing other PPH fractions had larger droplet sizes (D [4,3] = 1.54–1.61 µm) on the first day of storage. The significant increase in droplet size when 33% of TW20 was replaced with fractionated hydrolysates suggests a potential competition between TW20 and the fractionated PPHs for adsorption at the interface. A study performed with PPHs and TW20 by Cheng et al. [30] showed that PPHs were partitioned into the interfacial membrane, and they found that combinations of PPHs and TW20 provided more uniformly distributed oil droplets compared to oil droplets obtained when TW20 was used alone. Independent of the sizes of the fractions, the fractions changed the characteristics of the interface, affecting the droplet size and surface charge. TW20 stabilizes the droplets mainly due to steric repulsion [20], and therefore, the charge of the PPH fractions as well as their partitioning at the interface might have a role in that change. Zhang et al. [31] demonstrated the critical role of interfacial characteristics in the oxidative stability of emulsions co-stabilized with TW20 and pea protein hydrolysates. Their findings showed that TW20 alone did not form a defined interfacial membrane, while its combination with protein hydrolysates led to a thin, discontinuous layer due to peptide unfolding and competitive adsorption. Notably, the presence of TW20 led to the partial or complete displacement of the proteins from the droplet surfaces, thereby influencing the emulsions’ susceptibility to oxidation. Similarly, Yi et al. [32] observed that in walnut oil-in-water emulsions, sodium caseinate proteins were competitively displaced by TW20. The degree of displacement varied with TW20 concentration and affected lipid and protein co-oxidation by altering the ratio of adsorbed to free protein content in the system. Yu et al. [33] also reported competitive adsorption behavior in emulsions stabilized with casein hydrolysates and TW20. Although the present study did not include interfacial tension or adsorption kinetics measurements, such analyses could provide deeper insight into the dynamic partitioning and cooperative adsorption mechanisms underlying the observed synergistic effects between PPHs and TW20.

The interaction between DATEM and PPH fractions was different; smaller fractions (<0.8 kDa and 0.8–5 kDa) interfered less with the ability of DATEM to provide good stability. This might be due to strong electrostatic forces provided by DATEM, which were not greatly affected by the incorporation of the hydrolysates, as the emulsions had high absolute values in terms of zeta potential (<−78 ± 3.07 mV). Surface charges higher than ±30 mV are considered good for preventing oil droplet aggregation through electrostatic repulsion [34]. Although TW20 emulsions had absolute zeta values lower than 30 mV, they were physically stable due to the rapid movement of TW20 to the oil–water interface during homogenization [35]. In addition, zeta potential is one of the factors that affects the physical stability of emulsions. While higher absolute zeta potential values are generally associated with increased electrostatic repulsion and improved stability, they do not alone guarantee long-term physical stability, as previously observed [36].

### 3.3. Oxidative Stability

Besides providing physical stability due to their emulsifying properties, PPHs can also prevent lipid oxidation in oil-in-water emulsions [14,23]. Therefore, the oxidative stability of emulsions prepared with unfractionated PPH and different fractions of PPH in combination with DATEM or TW20 were evaluated in terms of primary indications of lipid oxidation—PV and consumption of tocopherols—and in terms of secondary oxidation products—the formation of volatile compounds.

#### 3.3.1. Formation of Hydroperoxides

**Figure 2:**
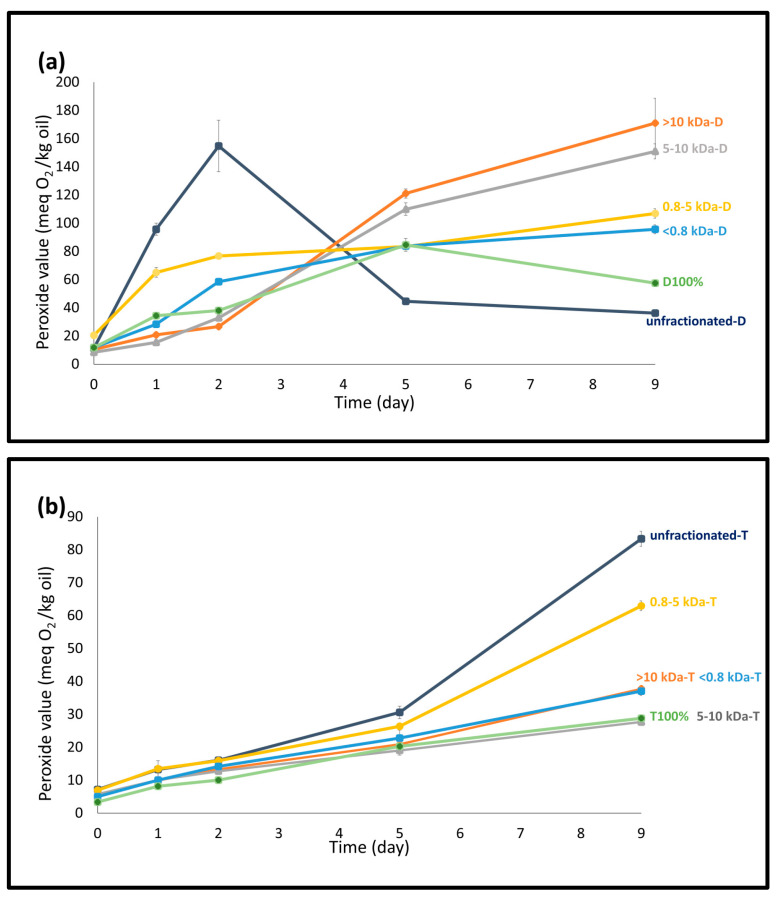
Peroxide value (PV) determined in 5 wt% fish-oil-in-water emulsions stabilized with (**a**) DATEM and (**b**) Tween 20 and containing the different fractions of potato protein hydrolysates (<0.8 kDa to >10 kDa) and unfractionated potato protein hydrolysate during 9 days of storage. (D and T demonstrate the DATEM and Tween 20, respectively and indicate the surfactant used in the emulsion.)

Figure 2 shows the peroxide value (PV) results of the emulsions, while detailed statistical comparisons (*p* < 0.05) are provided in Table S2 of the Supplementary Materials. On the day of production (day 0), emulsions prepared with DATEM showed PVs ranging from 8.50 ± 0.13 to 20.58 ± 1.17 meq O_2_/kg oil, whereas emulsions with TW20 ranged from 3.40 ± 0.32 to 7.19 ± 0.26 meq O_2_/kg oil, both notably higher than fresh oil’s PV (0.31 ± 0.01 meq O_2_/kg oil). Such high initial PVs have also been reported previously [36] and were attributed to processing conditions, particularly the homogenization step, which can promote lipid oxidation [37]. Among the DATEM emulsions, the 0.8–5 kDa-D emulsion showed the highest initial PV (20.58 ± 1.17 meq O_2_/kg oil). Although the D100% emulsion appeared physically stable, it exhibited a rapid increase in PV during storage, likely due to pre-existing oxidation in DATEM itself (PV = 11.58 ± 1.81 meq O_2_/kg oil). This pre-oxidation could explain the sharp rise in PV for all DATEM emulsions during storage, with values exceeding the method’s accuracy limit (40 meq/kg). Additionally, the higher negative surface charges observed in DATEM emulsions may have promoted the attraction of metal ions, further accelerating the lipid oxidation in those emulsions [38]. During storage, PV increased progressively in all DATEM emulsions except unfractionated-D and D100%. In the case of unfractionated-D, PV peaked at 154.83 meq O_2_/kg oil on day 2, followed by a decline, suggesting hydroperoxide’s decomposition to secondary oxidation products. Similarly, D100% showed a PV decrease after day 5. In contrast, 0.8–5 kDa-D and <0.8 kDa-D emulsions exhibited continuous PV increases throughout storage, reaching 106.83 ± 3.35 and 95.70 ± 3.17 meq O_2_/kg oil, respectively, by the end of the study. These final values were significantly higher than that of unfractionated-D (36.33 ± 1.56 meq O_2_/kg oil), further supporting the likelihood of hydroperoxide decomposition in the unfractionated-D starting from day 2.

For the emulsions prepared with TW20, most samples, except for unfractionated-T and 0.8–5 kDa-T, maintained PVs below 40 meq O_2_/kg oil and showed a progressive trend with a small slope throughout the storage. However, unfractionated-T and 0.8–5 kDa-T showed a sharp increase after day 5, reaching 83.32 ± 2.28 and 62.99 ± 1.48 meq O_2_/kg oil, respectively, by the end of the storage period. Among all TW20 emulsions, the 5–10 kDa-T (27.71 meq O_2_/kg oil) together with T100% (28.84 ± 0.21 meq O_2_/kg oil) were the least oxidized at the end of the storage when evaluated by their PVs. The poor oxidative performance of 0.8–5 kDa-T and unfractionated-T was consistent with previous findings by Yesiltas, García-Moreno et al. [18], where both unfractionated PPH and its 0.8–5 kDa fraction were used as a standalone emulsifier in the system. Interestingly, the <0.8 kDa fraction was the worst in preventing the peroxidation in that study (>200 meq O_2_/kg oil), while in the present study, when combined with TW20, the same fraction showed much improved performance, reaching a PV with a value of 37.07 ± 1.34 meq O_2_/kg oil at the end of storage. This suggests that the interaction with TW20 may have contributed to enhanced oxidative protection. Furthermore, PPH fractions higher than 5 kDa have previously been shown to have good radical scavenging and metal chelating activities [18], which likely contributed to the improved oxidative stability observed in the 5–10 kDa-T and >10 kDa-T emulsions. Overall, despite the good physical stability observed in DATEM-containing emulsions (as indicated by smaller droplet sizes and more negative zeta potentials), their oxidative stability was compromised. This was likely due to the pre-oxidized state of DATEM itself, which had a relatively high initial PV. Previous studies have reported that the oxidation level of the emulsifiers prior to emulsion production can significantly affect the oxidation status of the emulsions [32,39]. Conversely, the fractions of 5–10 kDa, >10 kDa and <0.8 kDa showed good oxidative stability with TW20, suggesting their potential as clean-label co-emulsifiers with antioxidant properties.

#### 3.3.2. Consumption of Tocopherols

**Figure 3:**
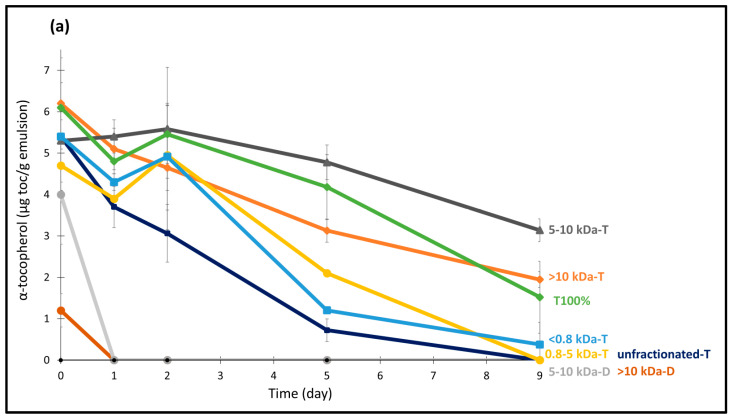

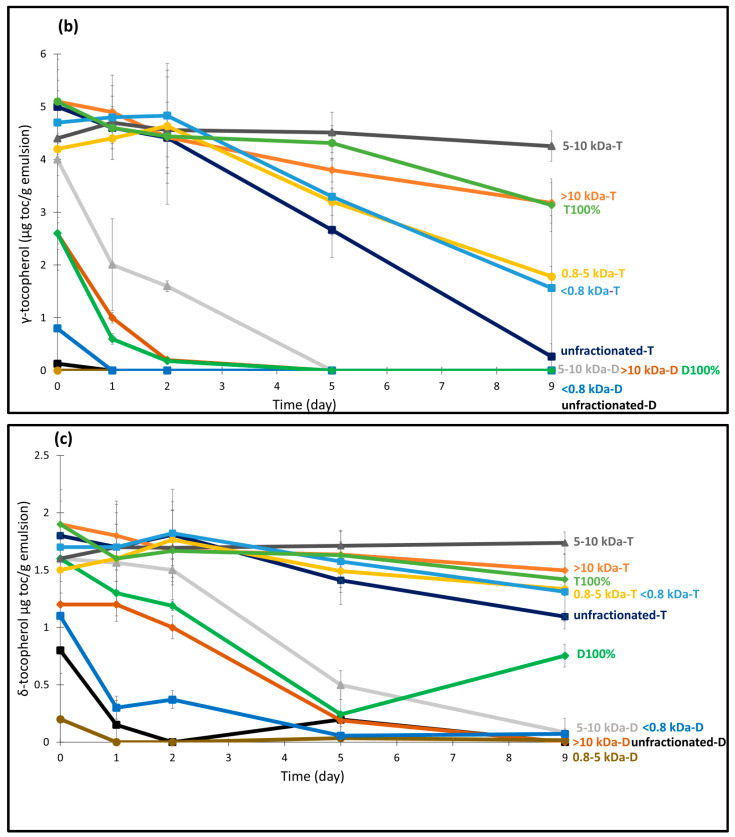
Consumption of (**a**) α-tocopherol, (**b**) γ-tocopherol, and (**c**) δ-tocopherol in 5 wt% fish oil in water stabilized with DATEM and Tween 20 and containing the different fractions of potato protein hydrolysates (<0.8 kDa to >10 kDa) or unfractionated potato protein hydrolysate during 9 days of storage. (D and T stands for DATEM and Tween 20, respectively. Emulsions not depicted in the legend lacked related tocopherols.)

Another indicator of oxidative stability is the change in the content of tocopherol, and this has been investigated by analyzing the three tocopherol homologues (alpha-, gamma-, and delta-tocopherol) present in fish oil during storage (Figure 3 and Table S3). Based on the initial concentration in the oil and 5 wt% oil content in the emulsions, the expected values were 8.8 ± 0.26 µg/g sample, 5.45 ± 0.16 µg/g sample, and 1.95 ± 0.06 µg/g sample for alpha-, gamma- and delta-tocopherol, respectively. However, already on day 0, all emulsions showed lower-than-expected tocopherol levels, which could be due to the consumption of tocopherols during the production process. Moreover, the tocopherol content of all the emulsions was rapidly depleted in the emulsions containing DATEM due to the high degree of oxidation. Among the homologues, α-tocopherol was the most rapidly consumed and was undetectable on day 0 in all DATEM containing emulsions, except for those with 5–10 kDa (4 ± 0.1 µg toc/sample) and >10 kDa (1.2 ± 0.4 µg toc/sample) fractions. This trend aligns with previous findings by Yeşiltaş et al. [36], where α-tocopherol was also quickly consumed in fish oil emulsions stabilized with various peptides and, attributed to its strong hydrogen-donating ability. For γ-tocopherol, the 5–10 kDa and >10 kDa fractions retarded its consumption for up to 5 days, while δ-tocopherol was almost depleted at the end of the storage in all DATEM emulsions. Despite this slower consumption of these tocopherol homologues, these emulsions had a fast and progressive increase in PV throughout the storage, as previously discussed (Section 3.3.1). It is important to note that PV is only one of the indicators of lipid oxidation. A higher PV does not necessarily indicate secondary oxidation, as hydrolysates with metal-chelating activity may prevent the metal-catalyzed decomposition of peroxides [18]. For example, Padial-Domínguez et al. [38] observed that increasing PV in soy protein hydrolysate-stabilized emulsions did not result in a higher concentration of volatiles.

In contrast to emulsions containing DATEM, all PPH fractions and unfractionated PPH helped to retard or prevent the consumption of tocopherols to varying extents in emulsions stabilized with TW20. Consistent with the PV results, the 5–10 kDa and >10 kDa fractions were the best ones in protecting tocopherol content during storage. Notably, the 5–10 kDa-T emulsion performed even better than the surfactant-alone emulsion, T100%, for conserving all three tocopherol homologues. Apart from a small decrease in α-tocopherol toward the end of storage, this emulsion maintained tocopherol levels close to those on day 0. In a previous study on emulsions with these fractions added as the sole emulsifiers, the 5–10 kDa fraction of PPH was found to have the best metal chelating activity compared to fractions of PPH [18]. Peptide properties such as flexibility, solubility, and hydrophobicity affect the peptides’ possible location and conformational changes at the interface and can affect their antioxidant potential. Thus, the metal chelating activity and molecular size of this fraction, 5–10 kDa, may have provided both chemical stability and a physical barrier that prevented or slowed down the oxidation process [18]. Although <0.8 kDa-T and 0.8–5 kDa-T were unable to protect α-tocopherol from depletion by the end of storage, they could protect γ- and δ -tocopherols from depletion during the storage. Interestingly, in some emulsions, such in 5–10 kDa-T and T100% for α-tocopherol and in 0.8–5 kDa-T for δ-tocopherol, tocopherol content tended to increase over time. This may be attributed to the regeneration of oxidized tocopherols by antioxidant peptides present in these hydrolysate fractions [40]. Although smaller fractions of PPH (<5 kDa) showed limited antioxidant activity and were ineffective in providing oxidative stability when used as sole emulsifiers in the system [18], combining them with TW20 in the current study enhanced both physical and oxidative stability.

#### 3.3.3. Development of Secondary Oxidation Volatile Compounds

**Figure 4:**
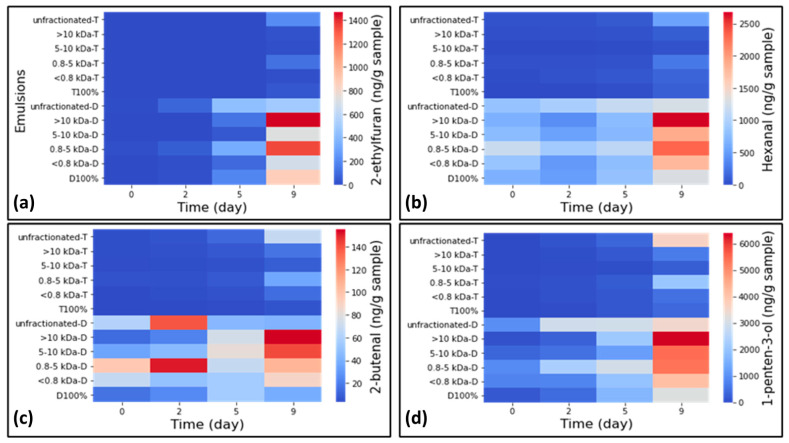
Formation of some secondary oxidation products in a 5 wt% fish-oil-in-water emulsion containing different fractions of potato protein hydrolysates (<0.8 kDa to >10 kDa) and unfractionated PPH and surfactants (DATEM and TW20) during the storage: (**a**) 2-ethylfuran, (**b**) hexanal, (**c**) 2-butenal, and (**d**) 1-penten-3-ol (D and T are DATEM and Tween 20, respectively, and indicate the surfactant used in the emulsion).

Hydroperoxides formed in the primary oxidation decompose to volatile compounds in the presence of prooxidants such as transition metals or by high temperature and result in off-flavors, which can be unacceptable to consumers [41]. Dynamic headspace coupled with GC-MS was used for the measurement of these secondary oxidation volatile compounds. Sixteen volatile compounds were identified and quantified (Table S4, Supplementary Materials). Figure 4 shows the change in levels of four of them, 2-ethyl furan, hexanal, 2-butenal and 1-penten-3-ol, during storage. 2-Ethylfuran and 1-penten-3-ol were selected because their concentration was higher at the end of the storage period than on day 0 (also observed for other volatiles *(E*,*E)*-2,4-heptadienal, 1-pentanol, pentanal, heptanal, *(Z)-*4-heptenal, *(E)-*2-heptenal, octanal, *(E*,*E)*-2,4-deceadienal, and 1-octen-3-ol), and 2-butenal and hexanal represented a trend also observed for other volatiles (1-penten-3-one, 2,3-pentanedione, *(E)*-2-hexenal).

Throughout the storage period, for TW20 emulsions, all PPH fractions (>10 kDa, 5–10 kDa, 0.8–5 kDa and <0.8 kDa), as well as the unfractionated hydrolysate, effectively inhibited the formation of 2-ethylfuran, maintaining levels comparable to TW20 alone, with a lag phase of 5 days (Figure 4a). In contrast, emulsions containing DATEM showed a sharp increase in the concentration of 2-ethylfuran between days 2 and 9, except for the unfractionated-D emulsion, which maintained relatively stable levels (439.3 ± 31.2 and 502.1 ± 58.8 ng/g on days 5 and 9, respectively). Generally, unfractionated-D, 5–10 kDa-D, <0.8 kDa-D, and D100% emulsions had significantly lower concentrations of this volatile compound during storage than the other DATEM-containing emulsions. Nonetheless, all DATEM emulsions, except for unfractionated-D, exhibited higher 2-ethylfuran levels on day 9 than those with TW20, regardless of peptide addition.

In the case of hexanal (Figure 4b), all DATEM emulsions already contained high levels of this volatile (671.9–1173.2 ng/g) on day 0, consistent with their higher initial PVs. Notably, the 0.8–5 kDa-D emulsion had a significantly higher hexanal concentration on day 0 than the other DATEM emulsions (*p* < 0.05). A slight decrease was observed during the first two days, followed by a marked increase after day 5 in all DATEM-containing emulsions, except for unfractionated-D. In contrast, TW20 emulsions showed low hexanal formation for all fractions, similar to T100%. However, unfractionated-T had the highest hexanal level by the end of storage (553.3 ± 38.9 ng/g). It is worth noting that, among these four volatiles, 1-penten-3-ol had the highest concentration during 9-day storage, consisting with the previous secondary lipid oxidation indicators in fish oils [18,36]. A similar trend was observed for the formation of 2-butenal and 1-penten-3-ol in TW20-containing emulsions (Figure 4c,d), where >10 kDa, 5–10 kDa and <0.8 kDa effectively reduced the formation of 1-penten-3-ol compared to unfractionated hydrolysate and 0.8–5 kDa fractions. This finding is consistent with a previous study by Yesiltas, García-Moreno et al. [18]. Overall, the 5–10 kDa fraction was the most effective one for oxidative stability in TW20 emulsions. For emulsions containing DATEM, the trend differed, with a steady increase in all volatiles during storage for >10 kDa-D and 5–10 kDa-D emulsions. In contrast, 0.8–5 kDa-D and unfractionated-D emulsions showed an initial increase in the first two days, followed by a decrease on day 5 and a subsequent rise towards the end of storage for 2-butenal formation. Notably, 1-penten-3-ol was abundant in all DATEM-containing emulsions, with >10 kDa-D showing the highest concentration.

Overall, combining the >10 kDa, 5–10 kDa, and <0.8 kDa fractions with TW20 resulted in secondary oxidation volatile levels comparable to those of the T100% emulsion throughout storage, indicating good oxidative stability. In contrast, emulsions with DATEM showed higher levels of volatiles, particularly in >10 kDa-D, 5–10 kDa-D, and 0.8–5 kDa-D emulsions, suggesting that the strong interactions observed in DATEM emulsions likely contributing to good physical stability, may have negatively impacted oxidative stability. For instance, highly negative zeta potential values observed in DATEM-containing emulsions may also have had an impact on oxidative stability as a very negative zeta potential could attract the Fe^+2^ ions to the interface and favor metal-catalyzed oxidation [32,42]. A smaller droplet size of DATEM-containing emulsions and thus a larger surface area compared to TW20 emulsions can also increase exposure to prooxidants, accelerating the rate of lipid oxidation [6,7]. Additionally, DATEM was already oxidized, which might have accelerated oxidation at the interface. Thereby, DATEM could have facilitated the oxidation of the hydrolysate fractions, further degrading the stability of the emulsion [32,42]. Although the <0.8 kDa fraction also supported good physical stability, it did not lead to elevated levels of volatile formation. This emphasizes the importance of exploring surfactant–peptide interactions and interfacial characteristics, including the structure of the interface, the partitioning of compounds (surfactants and peptides) and molecular interactions that critically influence oxidation [7].

In summary, volatile formation, PVs, and tocopherol content indicated that TW20 combined with 5–10 kDa, >10 kDa and <0.8 kDa fractions was effective in providing oxidative stability with 5–10 kDa-T, providing comparable performance to T100%. In contrast, DATEM-containing emulsions, including D100%, had higher volatile content compared to TW20 emulsions, and none of the fractions could prevent the formation of volatiles under the prevailing conditions. Among them, unfractionated-D performed slightly better at limiting the formation of 2-ethylfuran but did not significantly improve the oxidative outcomes for other volatiles or tocopherols. Neither the fractions nor the unfractionated hydrolysate could prevent the increase in PV during storage or the depletion of tocopherols in DATEM emulsions. Emulsions containing DATEM exhibited higher oxidation rates, likely because their increased interfacial area, strongly negative zeta potential, and potential prooxidant role of DATEM in these emulsions created a conducive environment for oxidation. The 5–10 kDa PPH with TW20 demonstrated better oxidative stability than other PPHs, potentially owing to its superior interfacial properties, including an effective decrease in surface tension, enhanced metal-chelating activity, and improved interface coverage, as shown in previous research [18]. This suggests that replacing 33% of the TW20 with 5–10 kDa PPH maintains similar performance to that of T100%, as indicated by comparable levels of peroxide and volatile formation and similar rates of tocopherol depletion. While combining PPHs with surfactants helped to address their limited emulsifying capacity, further optimization, such as hydrolysis parameters or incorporating other techniques, such as ultrasound or hydrostatic pressure, could enhance their dual functionality as standalone emulsifiers with antioxidant activity. For instance, ultrasound-microwave-assisted enzymatic hydrolysis improved the emulsifying properties of sweet PPHs, while high hydrostatic pressure enhanced their interfacial adsorption and antioxidant activity [43]. By enhancing their emulsifying and antioxidant characteristics, they can be used as sole emulsifiers in the formulations. Such advancements may make it feasible to replace synthetic surfactants in food emulsions completely, paving the way for clean-label formulations that use protein hydrolysates as effective, dual-function emulsifiers.

## 4. Conclusions

This study investigated the emulsifying and antioxidant performance of unfractionated and fractionated potato protein hydrolysates (PPHs) in combination with food-grade emulsifiers Tween 20 (TW20) and DATEM in 5 wt% fish-oil-in-water emulsions. The results demonstrated different interactions between the surfactants and PPHs. TW20-containing emulsions had competitive displacement between TW20 and PPHs. Regarding oxidative stability, emulsions with DATEM showed higher peroxide values (PV), faster tocopherol depletion, and increased formation of secondary oxidation volatiles compared to TW20 emulsions. However, the combination of PPH fractions with TW20 provided comparable oxidative stability to T100%, and the 5–10 kDa fraction was particularly effective in preventing tocopherol depletion. These findings highlight a promising strategy for developing clean-label emulsifiers from plant-based by-products with antioxidant properties. Nevertheless, the standalone emulsifying capacity of PPHs remains limited. Further studies should focus on understanding the interfacial behavior of PPHs, including their partitioning, orientation and interactions at the oil–water interface, to have a better understanding of underlying adsorption dynamics at the interface. Additionally, optimizing protein hydrolysate production conditions (e.g., hydrolysis parameters coupling enzymatic treatment with other techniques) may enhance both their emulsifying and antioxidant functionality in future applications.

## Figures and Tables

**Table 1:** Protein content of the fractionated PPHs.

Molecular Weight of the Potato Protein Hydrolysates	Protein %
Unfractionated	79.42 ± 1.94
>10 kDa	71.50 ± 3.09
5–10 kDa	79.79 ± 0.19
0.8–5 kDa	79.97 ± 0.58
<0.8 kDa	73.31 ± 4.46

**Table 2:** Volume-weighted mean diameter (D [4,3]), surface-weighted mean (D [3,2]), and zeta potential of the 5 wt% rapeseed oil-in-water emulsions ^1^ produced on a small scale (2 g) stabilized with either DATEM, Tween 20, and PPH or combinations of PPH with DATEM and Tween 20 on days 1 and day 7.

Emulsion Code *	D [4,3] (µm) (Day 1)	D [4,3] (µm) (Day 7)	D [3,2] (µm) (Day 1)	D [3,2] (µm) (Day 7)	Zeta Potential (mV) (Day1)
Unfractionated PPH100%	5.30 ± 0.56 ^E,a^	18.94 ± 2.50 ^D,b^	0.24 ± 0.02 ^C,a^	0.57 ± 0.05 ^E,b^	−56.1 ± 1.8 ^C^
D33%	1.31 ± 0.18 ^BC,a^	6.19 ± 0.75 ^C,b^	0.21 ± 0.01 ^BC,a^	0.36 ± 0.03 ^C,b^	−80.0 ± 2.7 ^B^
D50%	1.06 ± 0.20 ^BC,a^	3.94 ± 0.75 ^BC,b^	0.20 ± 0.01 ^AB,a^	0.29 ± 0.02 ^B,b^	−84.7 ± 1.9 ^B^
D67%	0.37 ± 0.02 ^A,a^	1.03 ± 0.04 ^A,b^	0.17 ± 0.01 ^A,a^	0.21 ± 0.01 ^A,b^	−88.3 ± 5.0 ^AB^
D100%	0.35 ± 0.03 ^A,a^	2.91 ± 0.50 ^AB,b^	0.17 ± 0.01 ^A,a^	0.18 ± 0.01 ^A,a^	−93.5 ± 5.3 ^A^
T33%	2.77 ± 0.15 ^D,a^	3.06 ± 0.36 ^AB,a^	0.45 ± 0.02 ^F,a^	0.43 ± 0.00 ^D,a^	−26.0 ± 2.0 ^D^
T50%	1.46 ± 0.22 ^C,a^	1.38 ± 0.24 ^AB,a^	0.37 ± 0.02 ^E,a^	0.36 ± 0.01 ^C,a^	−24.4 ± 1.7 ^D^
T67%	0.96 ± 0.08 ^ABC,a^	0.88 ± 0.04 ^A,a^	0.33 ± 0.02 ^D,a^	0.31 ± 0.01 ^BC,a^	−20.4 ± 1.5 ^D^
T100%	0.79 ± 0.01 ^AB,a^	0.74 ± 0.01 ^A,a^	0.30 ± 0.00 ^D,a^	0.30 ± 0.00 ^BC,a^	−18.2 ± 0.8 ^D^

^1^ Mean ± standard deviation (n = 3). * Unfractionated PPH100% includes only unfractionated potato protein hydrolysate (PPH) as an emulsifier; in other emulsions, percentages show the weight percentage of the surfactant in the emulsion, and the rest is PPH. D and T stand for the emulsions containing DATEM and Tween 20, respectively. ^A–F^ Letters indicate significant differences between samples within the same day (ANOVA, Tukey’s post hoc test, *p* < 0.05). ^a,b^ Letters indicate significant changes during storage (ANOVA, Tukey’s post hoc test, *p* < 0.05).

**Table 3:** Volume-weighted mean (D [4,3]) and zeta potential of the 5 wt% fish-oil-in-water emulsions ^1^ produced on a large-scale (250 g) and stabilized the combinations of 33% *w*/*w* fractionated (<0.8 kDa to >10 kDa) or unfractionated potato protein hydrolysates with 67% *w*/*w* DATEM, Tween 20, and DATEM and Tween 20 alone (100%) on days 1 and 9.

Emulsion Code *	D [4,3] (µm) (Day 1)	D [4,3] (µm) (Day 9)	Zeta Potential (mV) (Day1)
unfractionated-D	1.189 ± 0.002 ^F,a^	1.309 ± 0.022 ^CD,b^	−78.00 ± 3.07 ^B^
>10 kDa-D	2.429 ± 0.038 ^I,a^	2.508 ± 0.004 ^F,b^	−84.78 ± 5.38 ^AB^
5–10 kDa-D	0.764 ± 0.002 ^D,a^	0.974 ± 0.059 ^B,b^	−85.05 ± 3.75 ^AB^
0.8–5 kDa-D	0.389 ± 0.002 ^C,a^	0.472 ± 0.001 ^A,b^	−78.43 ± 3.72 ^B^
<0.8 kDa-D	0.352 ± 0.000 ^BC,a^	0.354 ± 0.004 ^A,a^	−83.26 ± 5.55 ^B^
D100%	0.279 ± 0.005 ^A,a^	0.276 ± 0.002 ^A,a^	−94.08 ± 5.70 ^A^
unfractionated-T	1.051 ± 0.015 ^E,b^	0.947 ± 0.005 ^B,a^	−29.03 ± 2.03 ^C^
>10 kDa-T	1.607 ± 0.021 ^H,a^	1.575 ± 0.118 ^E,a^	−19.15 ± 0.80 ^CD^
5–10 kDa-T	1.541 ± 0.011 ^G,b^	1.264 ± 0.071 ^C,a^	−18.58 ± 1.59 ^D^
0.8–5 kDa-T	1.604 ± 0.003 ^H,b^	1.501 ± 0.012 ^DE,a^	−16.93 ± 0.58 ^D^
<0.8 kDa-T	1.599 ± 0.003 ^H,a^	1.436 ± 0.173 ^CDE,a^	−19.80 ± 2.45 ^CD^
T100%	0.317 ± 0.001 ^AB,a^	0.330 ± 0.004 ^A,b^	−14.73 ± 1.61 ^D^

^1^ Mean ± standard deviation (*n* = 3). * D and T signify the DATEM and Tween20, respectively. Molecular weights show the corresponding fraction of potato protein hydrolysate used in the emulsion. ^A–I^ Letters indicate significant differences between samples within the same day (ANOVA, Tukey’s post hoc test, *p* < 0.05). ^a,b^ Letters indicate significant changes during storage (ANOVA, Tukey’s post hoc test, *p* < 0.05).

## Data Availability

The original contributions presented in this study are included in the article/Supplementary Material. Further inquiries can be directed to the corresponding author.

## Supplementary Materials

The following supporting information can be downloaded at https://www.mdpi.com/article/10.3390/foods14111974/s1. Figure S1: Physical stability of 5 wt% fish-oil-in-water emulsions stabilized with (a) DATEM and (b) Tween 20, containing various potato protein hydrolysate fractions (<0.8 kDa to >10 kDa) and the unfractionated hydrolysate, monitored over 9 days of storage. From left to right, emulsions containing unfractionated hydrolysate, >10 kDa, 5–10 kDa, 0.8–5 kDa and <0.8 kDa fractions, each combined with the respective surfactant, followed by emulsions prepared with 100% surfactant alone; Figure S2: Particle size distributions of 5 wt% fish-oil-in-water emulsions containing various potato protein hydrolysate fractions (<0.8 kDa to >10 kDa) and the unfractionated hydrolysate, stabilized with (a) DATEM on day 1, (b) DATEM on day 9, and (c) Tween 20 on day 1, and (d) on day 9; Table S1: Surface-weighted mean D [3,2]) of the 5 wt% fish-oil-in-water emulsions produced on a large scale (250 g) stabilized with the combinations of 33% *w*/*w* fractionated (<0.8 kDa to >10 kDa) or unfractionated potato protein hydrolysates with 67% *w*/*w* DATEM, Tween 20, and DATEM and Tween 20 alone (100%) on days 1 and 9; Table S2: Peroxide values (PV, expressed as meq O_2_/kg oil) for 5 wt% fish-oil-in-water emulsions stabilized with combinations of fractionated (<0.8 kDa to >10 kDa) and unfractionated potato protein hydrolysates with DATEM, Tween 20, and DATEM and Tween 20 alone; Table S3: Tocopherol contents of 5 wt% fish-oil-in-water emulsions stabilized with combinations of fractionated (<0.8 kDa to >10 kDa) and unfractionated potato protein hydrolysates with DATEM, Tween 20, and DATEM and Tween 20 alone; Table S4: Volatile compound contents of the 5 wt% fish-oil-in-water emulsions stabilized with the combinations of fractionated (<0.8 kDa to >10 kDa) and unfractionated potato protein hydrolysates with DATEM, Tween 20, and DATEM and Tween 20 alone.

## Abbreviations

The following abbreviations are used in this manuscript:

PUFAs	Polyunsaturated Fatty Acids
DATEM	Diacetyl Tartaric Acid Ester of Mono- and Diglycerides
PPH	Potato Protein Hydrolysate
PV	Peroxide Value
TSI	Turbiscan Stability Index
TW20	Tween 20
