# Supplementary material for: Effect of Combining Surfactants with Potato Protein Hydrolysates on Their Emulsifying and Antioxidant Properties in Fish-Oil-in-Water Emulsions
Source: Foods. 2025 Jun 2;14(11):1974. doi: 10.3390/foods14111974 (PMC12155317; doi:10.3390/foods14111974)
Supplement: Supplementary file 1 [file foods-14-01974-s001.zip › foods-3650424-supplementary.pdf]

Supplementary Materials

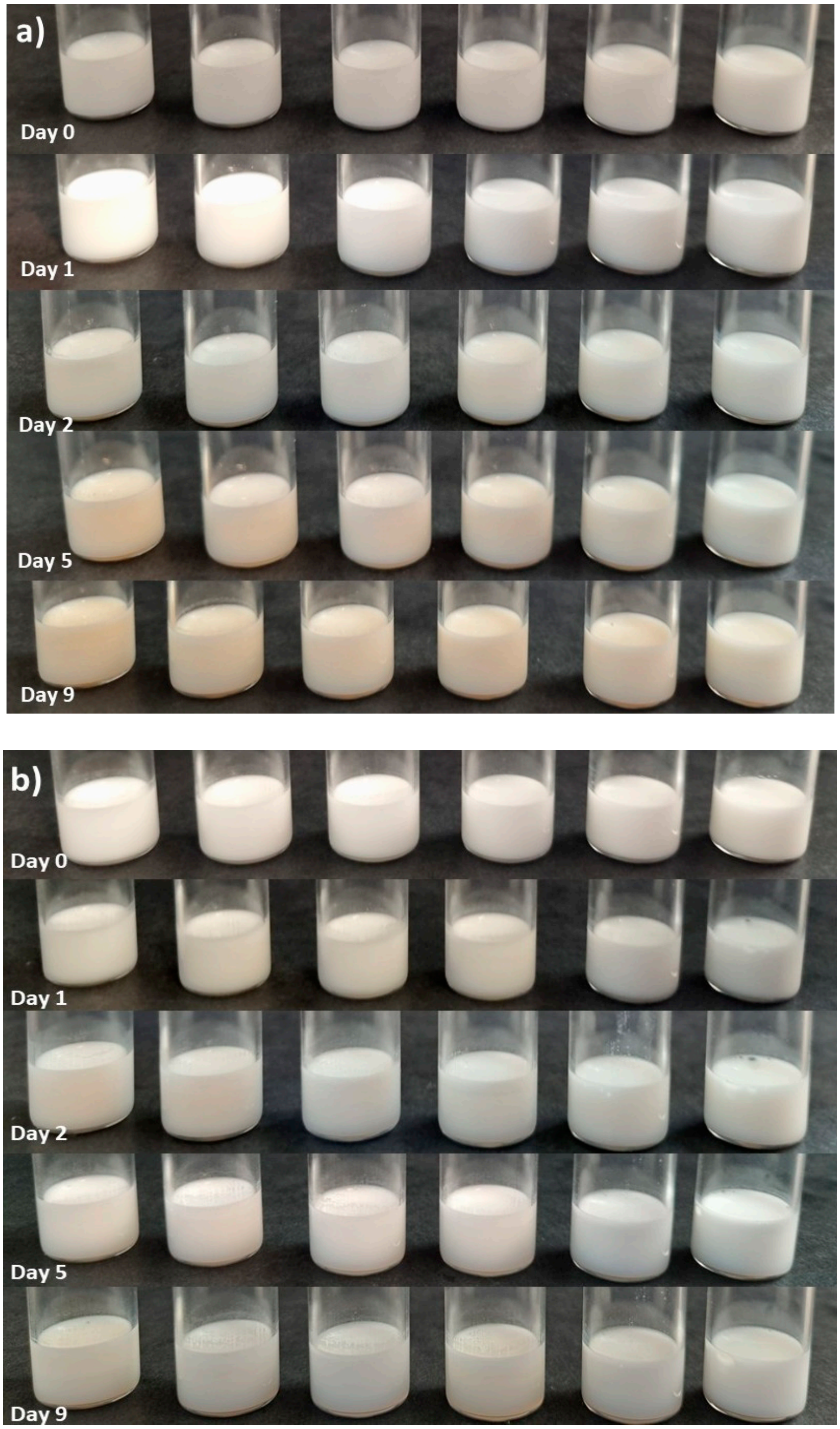

Figure S1. Physical stability of 5 wt% fish oil-in-water emulsions stabilized with a) DATEM and b) Tween 20, containing various potato protein hydrolysate fractions (<0.8 kDa to >10 kDa) and the unfractionated hydrolysate, monitored over 9 days of storage. From left to right emulsions containing unfractionated hydrolysate, >10 kDa, 5-10 kDa, 0.8-5 kDa and <0.8 kDa

fractions, each combined with the respective surfactant, followed by emulsions prepared with 100% surfactant alone.

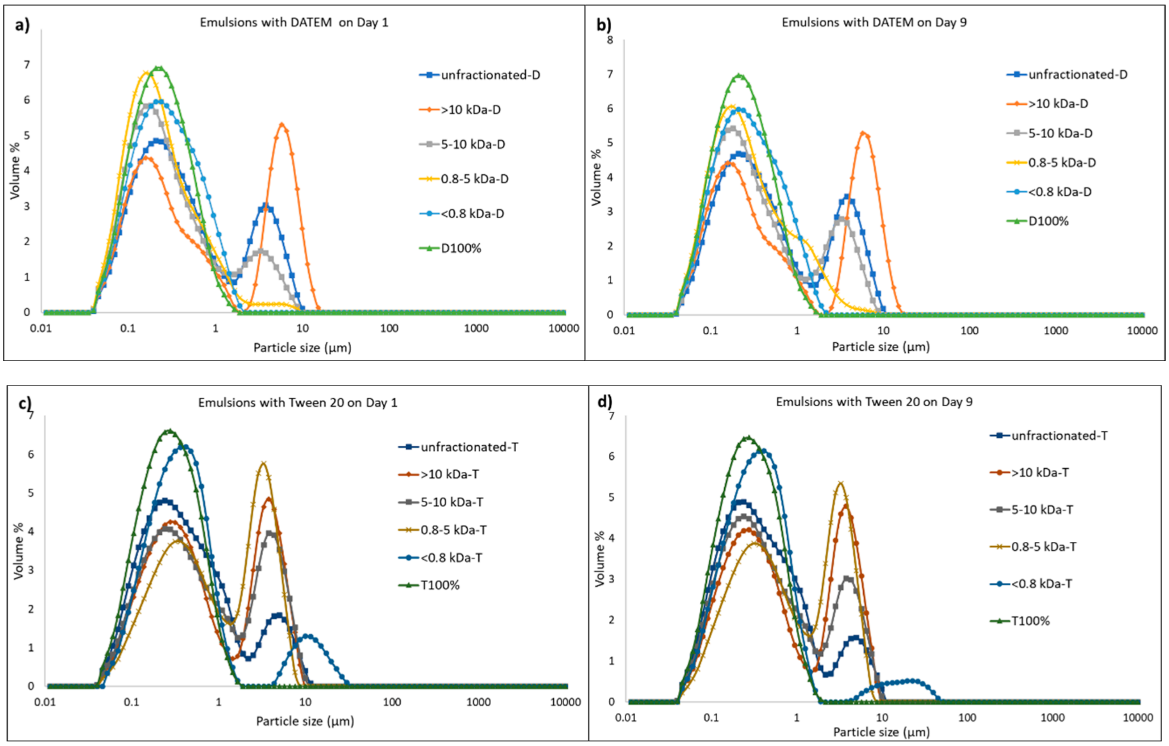

Figure S2. Particle size distributions of 5 wt% fish oil-in-water emulsions containing various potato protein hydrolysate fractions (<0.8 kDa to >10 kDa) and the unfractionated hydrolysate, stabilized with: a) DATEM on day 1, b) DATEM on day 9, and c) Tween 20 on day 1, and d) on day 9.

Table S1. Surface-weighted mean D [3,2]) of the 5 wt% fish oil-in-water emulsions[1] produced in large-scale (250 g) stabilized with the combinations of 33 % w/w fractionated (<0.8 kDa to >10 kDa) or unfractionated potato protein hydrolysates with 67 % w/w DATEM, Tween 20, and DATEM and Tween 20 alone (100%) on days 1 and 9.

D [3,2] (µm) (Day 1)	D [3,2] (µm) (Day 9)
0.224±0.000^{D,a}	0.237±0.006^{DE,b}
0.238±0.005^{E,a}	0.237±0.001^{DE,a}
0.178±0.001^{C,a}	0.200±0.011^{C,b}
0.154±0.001^{A,a}	0.172±0.001^{AB,b}
0.182±0.001^{C,a}	0.181±0.002^{AB,a}
0.165±0.004^{B,a}	0.163±0.002^{A,a}
0.231±0.004^{DE,b}	0.223±0.002^{D,a}
0.292±0.005^{H,a}	0.282±0.016^{F,a}
0.280±0.003^{G,b}	0.249±0.009^{E,a}
0.363±0.001^{I,b}	0.336±0.001^{G,a}
0.254±0.001^{F,b}	0.228±0.003^{D,a}

0.183±0.000^{C,a}	0.187±0.003^{BC,b}

[1] Mean± standard deviation (n=3).

[*] D and T signify the DATEM and Tween20, respectively. Molecular weights show the corresponding fraction of potato protein hydrolysate used in the emulsion.

[A-I] Letters indicate significant differences between samples within the same day (ANOVA, Tukey's post-hoc test, p<0.05).

[a-b] Letters indicate significant changes during storage (ANOVA, Tukey's post-hoc test, p<0.05).

Table S2. Peroxide values (PV, expressed as meq O_2/kg oil) for 5% fish oil-in-water emulsions[1] stabilized with the combinations of fractionated (<0.8 kDa to >10 kDa) and unfractionated potato protein hydrolysates with DATEM, Tween 20, and DATEM and Tween 20 alone.

Emulsion code[*]	Day 0	Day 1	Day 2	Day 5	Day 9
**unfractionated-D**	11.24±1.38^{D,a}	95.77±4.21^{F,b}	154.83±18.22^{F,c}	44.65±2.34^{C,a}	36.33±1.56^{AB,a}
**>10 kDa-D**	10.47±0.19^{CD,a}	20.91±0.86^{BC,a}	26.73±1.02^{ABC,a}	121.07±3.27^{F,b}	170.97±17.73^{G,c}
**5-10 kDa-D**	8.50±0.13^{BCD,a}	15.46±0.47^{AB,a}	32.87±1.37^{BC,b}	109.96±4.47^{E,c}	150.95±5.41^{G,d}
**0.8-5 kDa-D**	20.58±1.17^{E,a}	65.12±3.49^{E,b}	76.74±0.19^{E,c}	83.54±2.01^{D,c}	106.83±3.35^{F,d}
**<0.8 kDa-D**	11.81±0.40^{D,a}	28.30±0.56^{CD,b}	58.54±0.15^{DE,c}	83.74±1.56^{D,d}	95.7±3.17^{EF,e}
**D100%**	11.82±0.58^{D,a}	34.39±0.32^{D,b}	38.09±0.94^{CD,b}	84.6±4.54^{D,d}	57.47±1.26^{BC,c}
**unfractionated-T**	7.19±0.26^{BC,a}	13.25±0.79^{A,b}	16.04±0.45^{AB,b}	30.6±1.91^{B,c}	83.32±2.28^{DE,d}
**>10 kDa-T**	5.41±2.23^{AB,a}	9.96±0.56^{A,ab}	13.33±0.91^{AB,b}	20.9±0.17^{AB,c}	37.78±0.62^{AB,d}
**5-10 kDa-T**	5.77±0.68^{AB,a}	10.09±0.12^{A,b}	12.77±0.55^{AB,b}	19.05±1.41^{A,c}	27.71±0.00^{A,d}
**0.8-5 kDa-T**	6.94±0.21^{BC,a}	13.46±2.47^{AB,b}	15.92±0.01^{AB,b}	26.37±0.31^{AB,c}	62.99±1.48^{CD,d}
**<0.8 kDa-T**	5.07±0.17^{AB,a}	10.06±2.14^{A,ab}	14.22±0.56^{AB,b}	22.81±2.43^{AB,c}	37.07±1.34^{AB,d}
**T100%**	3.40±0.32^{A,a}	8.20±0.38^{A,b}	10.07±0.06^{A,b}	20.34±1.24^{A,c}	28.84±0.21^{A,d}

[1]Mean±standard deviation (n=2).

[*]D and T demonstrate the DATEM and Tween 20 respectively and indicate the surfactant and molecular weights show the corresponding fraction of potato protein hydrolysate used in the emulsion.

[A-G] Letters indicate significant differences between samples within the same day (ANOVA, Tukey's post-hoc test, p<0.05).

[a-e] Letters indicate significant changes in PV during storage (ANOVA, Tukey's post-hoc test, p<0.05).

Table S3. Tocopherol contents of 5% fish oil-in-water emulsions[1] stabilized with the combinations of fractionated (<0.8 kDa to >10 kDa) and unfractionated potato protein hydrolysates with DATEM, Tween 20, and DATEM and Tween 20 alone.

Tocopherols (μg/g sample)	Emulsion code*	Day 0	Day 1	Day 2	Day 5	Day 9
Alpha tocopherol	**unfractionated-D**	0.00^{A,a}	0.00^{A,a}	0.00^{A,a}	0.00^{A,a}	0.00^{A,a}
	**>10 kDa-D**	1.20±0.40^{AB,b}	0.00^{A,a}	0.00^{A,a}	0.00^{A,a}	0.00^{A,a}
	**5-10 kDa-D**	4.00±0.10^{ABC,b}	0.00^{A,a}	0.00^{A,a}	0.00^{A,a}	0.00^{A,a}
	**0.8-5 kDa-D**	0.00^{A,a}	0.00^{A,a}	0.00^{A,a}	0.00^{A,a}	0.00^{A,a}
	**<0.8 kDa-D**	0.00^{A,a}	0.00^{A,a}	0.00^{A,a}	0.00^{A,a}	0.00^{A,a}
	**D100%**	0.00^{A,a}	0.00^{A,a}	0.00^{A,a}	0.00^{A,a}	0.00^{A,a}
	**unfractionated-T**	5.40±0.40^{BC,c}	3.70±0.50^{B,bc}	3.06±0.70^{AB,b}	0.72±0.27^{AB,a}	0.00^{A,a}
	**>10 kDa-T**	6.20±1.10^{C,b}	5.10±0.50^{DE,ab}	4.65±1.50^{B,ab}	3.13±0.28^{DE,ab}	1.95±0.19^{CD,a}
	**5-10 kDa-T**	5.30±1.40^{BC,a}	5.40±0.40^{E,a}	5.58±1.49^{B,a}	4.78±0.42^{F,a}	3.14±0.28^{D,a}
	**0.8-5 kDa-T**	4.70±0.40^{BC,c}	3.90±0.01^{BC,c}	4.95±0.55^{B,c}	2.10±0.09^{CD,b}	0.00^{A,a}
	**<0.8 kDa-T**	5.40±1.30^{BC,c}	4.30±0.20^{BCD,bc}	4.92±1.29^{B,c}	1.20±0.05^{BC,ab}	0.38±0.53^{AB,a}
	**T100%**	6.10±3.30^{C}	4.80±0.30^{CDE}	5.46±0.70^{B}	4.18±0.79^{EF}	1.52±0.87^{BC}
Gamma tocopherol	**unfractionated-D**	0.13±0.04^{A,b}	0.00^{A,a}	0.00^{A,a}	0.00^{A,a}	0.00^{A,a}
	**>10 kDa-D**	2.60±0.20^{ABC,c}	1.00±0.10^{AB,b}	0.2±0.03^{A,a}	0.00^{A,a}	0.00^{A,a}
	**5-10 kDa-D**	4.00±0.10^{BC,c}	2.01±0.90^{B,b}	1.6±0.10^{A,b}	0.00^{A,a}	0.00^{A,a}
	**0.8-5 kDa-D**	0.00^{A,a}	0.00^{A,a}	0.00^{A,a}	0.00^{A,a}	0.00^{A,a}
	**<0.8 kDa-D**	0.80±1.10^{AB,a}	0.00^{A,a}	0.00^{A,a}	0.00^{A,a}	0.00^{A,a}
	**D100%**	2.60±0.30^{ABC,c}	0.60±0.10^{AB,b}	0.18±0.02^{A,ab}	0.00^{A,a}	0.00^{A,a}
	**unfractionated-T**	5.00±0.30^{C,c}	4.60±0.60^{C,bc}	4.41±0.67^{B,bc}	2.66±0.52^{B,b}	0.26±0.24^{A,a}
	**>10 kDa-T**	5.10±0.80^{C,a}	4.90±0.50^{C,a}	4.42±1.27^{B,a}	3.80±0.23^{BCD,a}	3.18±0.38^{BC,a}
	**5-10 kDa-T**	4.40±1.10^{BC,a}	4.70±0.30^{C,a}	4.56±1.01^{B,a}	4.51±0.38^{D,a}	4.25±0.29^{C,a}
	**0.8-5 kDa-T**	4.20±0.30^{BC,c}	4.40±0.20^{C,c}	4.63±0.22^{B,c}	3.20±0.26^{BC,b}	1.78±0.19^{AB,a}
	**<0.8 kDa-T**	4.70±1.00^{C,a}	4.80±0.80^{C,a}	4.83±0.99^{B,a}	3.30±0.73^{BCD,a}	1.56±1.37^{AB,a}
	**T100%**	5.10±2.50^{C,a}	4.60±0.30^{C,a}	4.44±0.39^{B,a}	4.31±0.33^{CD,a}	3.13±0.50^{BC,a}
Delta tocopherol	**unfractionated-D**	0.80±0.20^{AB,b}	0.15±0.21^{A,ab}	0.00^{A,a}	0.20±0.28^{A,ab}	0.00^{A,a}
	**>10 kDa-D**	1.20±0.10^{AB,b}	1.20±0.10^{BC,b}	1.00±0.10^{BC,b}	0.19±0.03^{A,a}	0.00^{A,a}
	**5-10 kDa-D**	1.60^{B,b}	1.56±0.50^{C,b}	1.50±0.10^{C,b}	0.50±0.13^{A,a}	0.10±0.10^{A,a}
	**0.8-5 kDa-D**	0.20^{A,b}	0.00^{A,a}	0.00^{A,a}	0.03±0.02^{A,a}	0.00^{A,a}
	**<0.8 kDa-D**	1.10±0.50^{AB,b}	0.30±0.10^{AB,ab}	0.37±0.08^{AB,ab}	0.06±0.08^{A,a}	0.07±0.02^{A,a}
	**D100%**	1.60±0.20^{B,c}	1.30±0.10^{C,c}	1.19±0.04^{BC,bc}	0.24±0.01^{A,a}	0.75±0.10^{B,b}
	**unfractionated-T**	1.80±0.10^{B,b}	1.70±0.20^{C,ab}	1.81±0.22^{C,b}	1.41±0.21^{B,ab}	1.09±0.04^{BC,a}
	**>10 kDa-T**	1.90±0.30^{B,a}	1.80±0.20^{C,a}	1.67±0.43^{C,a}	1.64±0.11^{B,a}	1.50±0.14^{CD,a}
	**5-10 kDa-T**	1.60±0.30^{B,a}	1.70±0.20^{C,a}	1.69±0.33^{C,a}	1.71±0.12^{B,a}	1.74±0.10^{D,a}
	**0.8-5 kDa-T**	1.50±0.10^{AB,a}	1.60±0.20^{C,a}	1.76±0.06^{C,a}	1.49±0.14^{B,a}	1.33±0.06^{CD,a}
	**<0.8 kDa-T**	1.70±0.40^{B,a}	1.70±0.40^{C,a}	1.82±0.38^{C,a}	1.58±0.27^{B,a}	1.31±0.33^{CD,a}
	**T100%**	1.90±0.80^{B,a}	1.60±0.10^{C,a}	1.67±0.13^{C,a}	1.63±0.07^{B,a}	1.42±0.01^{CD,a}

[1]Mean±standard deviation (n=2).

[*]D and T demonstrate the DATEM and Tween 20 respectively and indicate the surfactant and molecular weights show the corresponding fraction of potato protein hydrolysate used in the emulsion.

[A-F] Letters indicate significant differences between samples within the same day (ANOVA, Tukey's post-hoc test, p<0.05).

[a-c] Letters indicate significant changes in tocopherol contents during storage (ANOVA, Tukey's post-hoc test, p<0.05).

Table S4. Volatile compound contents of the 5% fish oil-in-water emulsions[1] stabilized with the combinations of fractionated (<0.8 kDa to >10 kDa) and unfractionated potato protein hydrolysates with DATEM, Tween 20, and DATEM and Tween 20 alone.

Volatile compounds (ng/g)	Emulsion code[*]	Day 0	Day 2	Day 5	Day 9
2-ethyl furan	unfractionated-T	0.00^{A,a}	0.00^{A,a}	5.10±3.13^{A,a}	213.84±14.72^{AB,b}
	>10 kDa-T	0.00^{A,a}	0.00^{A,a}	0.73±1.27^{A,a}	27.65±4.80^{A,b}
	5-10 kDa-T	0.00^{A,a}	0.00^{A,a}	0.00^{A,a}	11.64±4.54^{A,b}
	0.8-5 kDa-T	0.00^{A,a}	0.00^{A,a}	3.28±5.69^{A,a}	122.19±6.92^{A,b}
	<0.8 kDa-T	0.00^{A,a}	0.00^{A,a}	0.00^{A,a}	14.33±3.53^{A,b}
	T100%	0.00^{A,a}	0.00^{A,a}	0.00^{A,a}	34.44±6.49^{A,b}
	unfractionated-D	6.79±3.23^{B,a}	90.39±22.57^{C,a}	439.26±31.23^{F,b}	502.08±58.78^{BC,b}
	>10 kDa-D	0.00^{A,a}	0.23±0.39^{A,a}	134.99±22.14^{CD,b}	1465.85±95.66^{E,c}
	5-10 kDa-D	0.00^{A,a}	1.25±1.26^{A,a}	45.24±8.89^{AB,a}	728.14±119.10^{CD,b}
	0.8-5 kDa-D	12.48±3.70^{C,a}	59.07±6.61^{B,a}	346.38±48.94^{E,a}	1343.71±330.33^{E,b}
	<0.8 kDa-D	0.00^{A,a}	15.81±5.20^{A,a}	98.61±27.07^{BC,a}	676.99±114.69^{CD,b}
	D100%	0.00^{A,a}	20.62±5.37^{A,a}	196.07±39.74^{D,b}	866.87±90.69^{D,c}
2-butenal	unfractionated-T	4.96±0.29^{A,a}	6.61±0.40^{A,a}	13.40±0.97^{A,b}	68.69±3.29^{DE,c}
	>10 kDa-T	5.02±0.26^{A,a}	5.39±0.36^{A,a}	7.93±1.41^{A,b}	17.27±0.36^{ABC,c}
	5-10 kDa-T	4.44±0.43^{A,a}	5.14±0.21^{A,a}	5.78±0.79^{A,a}	9.67±1.55^{AB,b}
	0.8-5 kDa-T	6.15±0.04^{A,a}	5.67±0.31^{A,a}	8.63±1.58^{A,a}	36.93±2.53^{BC,b}
	<0.8 kDa-T	4.16±0.23^{A,a}	5.03±0.11^{A,a}	6.31±0.32^{A,a}	14.86±1.90^{ABC,b}
	T100%	3.60±0.12^{A,a}	3.89±0.26^{A,ab}	4.53±0.39^{A,b}	6.62±0.36^{A,c}
	unfractionated-D	61.35±26.74^{CD,a}	138.74±5.12^{D,b}	45.18±3.08^{B,a}	42.48±1.77^{CD,a}
	>10 kDa-D	14.45±1.00^{AB,a}	23.02±4.42^{B,a}	73.83±5.97^{CD,b}	155.13±4.45^{G,c}
	5-10 kDa-D	36.11±7.48^{BC,a}	46.24±5.62^{C,a}	82.52±10.11^{D,b}	141.57±17.17^{G,c}
	0.8-5 kDa-D	96.85±15.42^{E,a}	151.38±7.82^{D,b}	68.86±7.93^{CD,a}	106.48±24.60^{F,a}
	<0.8 kDa-D	68.53±14.54^{DE,ab}	50.04±8.74^{C,a}	56.75±18.72^{BC,ab}	89.15±11.03^{EF,b}
	D100%	16.69±2.11^{AB,a}	25.49±3.70^{B,a}	56.41±8.89^{BC,c}	39.33±3.37^{C,b}
1-penten-3-ol	unfractionated-T	17.72±8.02^{A,a}	127.44±15.03^{A,b}	386.75±31.04^{A,c}	3642.12±73.29^{CD,d}
	>10 kDa-T	2.36±2.23^{A,a}	55.96±5.40^{A,a}	178.62±57.67^{A,b}	679.76±37.56^{A,c}
	5-10 kDa-T	0.00^{A,a}	26.54±1.69^{A,a}	55.94±2.00^{A,a}	254.12±56.97^{A,b}
	0.8-5 kDa-T	26.67±0.57^{A,a}	78.14±16.53^{A,ab}	231.98±58.23^{A,b}	2078.84±119.47^{B,c}
	<0.8 kDa-T	13.18±2.95^{A,a}	82.37±12.16^{A,ab}	167.56±7.72^{A,b}	537.46±77.94^{A,c}
	T100%	10.26±1.57^{A,a}	38.64±7.24^{A,a}	156.05±38.27^{A,b}	410.70±15.68^{A,c}

	**unfractionated-D**	986.95±234.95^{C,a}	2913.86±44.65^{F,b}	2934.96±113.56^{D,b}	3509.09±147.10^{CD,c}
	**>10 kDa-D**	99.83±11.22^{A,a}	322.37±54.57^{B,a}	2169.69±197.09^{C,b}	6388.16±207.92^{F,c}
	**5-10 kDa-D**	388.83±101.25^{B,a}	536.44±106.70^{C,a}	1261.95±131.72^{B,b}	5423.90±344.64^{E,c}
	**0.8-5 kDa-D**	945.57±104.54^{C,a}	2324.75±76.43^{E,b}	2965.23±204.78^{D,b}	5343.55±882.67^{E,c}
	**<0.8 kDa-D**	808.51±181.59^{C,a}	836.32±90.56^{D,a}	1972.18±445.90^{C,b}	4127.30±357.43^{D,c}
	**D100%**	193.27±20.68^{AB,a}	457.00±32.03^{BC,a}	1705.83±162.78^{BC,b}	3181.57±118.87^{C,c}
**Hexanal**	**unfractionated-T**	50.40±9.80^{A,a}	43.40±4.30^{A,a}	87.60±5.40^{A,a}	553.30±38.90^{B,b}
	**>10 kDa-T**	25.00±5.80^{A,a}	31.40±3.40^{A,a}	43.50±12.00^{A,a}	112.90±5.10^{A,b}
	**5-10 kDa-T**	9.80±4.20^{A,a}	9.60±2.20^{A,a}	14.30±5.10^{A,a}	46.90±10.10^{A,b}
	**0.8-5 kDa-T**	38.70±0.50^{A,a}	33.90±3.10^{A,a}	51.10±9.80^{A,a}	261.40±9.20^{A,b}
	**<0.8 kDa-T**	20.10±2.90^{A,a}	44.20±3.50^{A,b}	63.00±2.10^{A,c}	148.80±4.40^{A,d}
	**T100%**	0.00^{A,a}	0.10±0.20^{A,a}	28.50±8.10^{A,b}	120.20±14.60^{A,c}
	**unfractionated-D**	835.90±279.30^{B,a}	963.30±26.20^{E,ab}	1154.20±23.60^{D,ab}	1268.10±83.20^{C,b}
	**>10 kDa-D**	673.70±41.30^{B,b}	415.20±34.90^{B,a}	766.00±18.30^{BC,b}	2674.40±64.80^{F,c}
	**5-10 kDa-D**	768.80±68.40^{B,b}	550.60±38.40^{D,a}	733.00±10.00^{B,b}	1881.90±94.20^{D,c}
	**0.8-5 kDa-D**	1173.20±77.00^{C,a}	914.10±6.80^{E,a}	1090.20±32.60^{D,a}	2315.40±289.60^{E,b}
	**<0.8 kDa-D**	851.4±75.50^{B,b}	489.10±32.30^{C,a}	788.50±96.00^{BC,b}	1793.60±32.90^{D,c}
	**D100%**	671.91±28.30^{B,b}	517.73±16.58^{CD,a}	834.18±37.34^{C,c}	1303.59±18.79^{C,d}
**1-penten-3-one**	**unfractionated-T**	7.50±1.40	17.90±3.30	41.20±3.50	358.40±65.20
	**>10 kDa-T**	4.10±0.90	7.50±0.20	24.70±6.60	68.60±3.60
	**5-10 kDa-T**	2.90±2.90	3.30±0.10	15.00±4.60	36.30±5.40
	**0.8-5 kDa-T**	7.90±0.70	11.90±0.70	26.90±7.30	194.30±42.5
	**<0.8 kDa-T**	2.60±0.20	9.80±1.40	19.10±5.40	48.00±2.60
	**T100%**	0.30±0.40	3.50±0.80	20.70±7.90	62.40±7.60
	**unfractionated-D**	173.90±28.00	313.50±48.80	68.60±11.80	70.30±5.60
	**>10 kDa-D**	32.50±5.50	62.30±10.80	214.50±57.00	312.40±9.50
	**5-10 kDa-D**	82.90±16.90	82.40±12.20	130.10±7.00	449.00±21.70
	**0.8-5 kDa-D**	173.90±23.20	241.90±2.40	158.70±51.90	160.30±29.30
	**<0.8 kDa-D**	139.20±26.20	119.70±11.70	159.20±37.00	192.40±11.90
	**D100%**	52.70±8.32	81.15±1.98	210.07±23.10	230.39±16.57
**Pentanal**	**unfractionated-T**	0.00	0.00	9.36±5.87	429.13±31.90
	**>10 kDa-T**	0.00	0.00	0.25±0.42	36.73±1.21
	**5-10 kDa-T**	0.00	0.00	0.00	0.28±0.49
	**0.8-5 kDa-T**	0.00	0.00	0.00	181.58±19.34
	**<0.8 kDa-T**	0.00	0.00	0.00	46.84±9.92
	**T100%**	0.00	0.00	0.00	32.37±6.22
	**unfractionated-D**	350.99±102.33	574.93±24.25	667.16±42.38	635.78±63.96
	**>10 kDa-D**	119.14±5.39	51.34±19.37	362.10±26.78	2005.08±47.09
	**5-10 kDa-D**	166.66±44.01	129.91±30.84	251.71±23.87	1374.76±136.89
	**0.8-5 kDa-D**	365.81±52.99	511.63±8.85	758.31±65.43	1669.17±311.41
	**<0.8 kDa-D**	309.36±75.57	311.44±40.03	562.11±143.46	1288.86±130.15
	**D100%**	81.17±22.11	98.06±9.83	318.62±41.27	697.47±40.58
**2,3 pentanedione**	**unfractionated-T**	79.20±20.00	211.20±12.90	545.80±15.40	853.50±225.60
	**>10 kDa-T**	61.50±15.80	108.10±6.30	209.70±68.70	675.50±40.40
	**5-10 kDa-T**	52.20±20.70	74.10±4.40	66.60±28.90	239.60±50.30
	**0.8-5 kDa-T**	114.50±10.80	103.60±19.40	220.00±91.30	815.10±24.60
	**<0.8 kDa-T**	79.20±12.40	91.70±11.00	145.20±7.30	534.60±85.50
	**T100%**	22.80±6.00	33.90±11.20	73.50±33.0	257.30±3.40
	**unfractionated-D**	827.80±193.40	1009.50±111.70	582.70±8.20	898.80±46.00
	**>10 kDa-D**	324.90±17.90	456.60±61.60	1069.90±12.10	747.10±30.50
	**5-10 kDa-D**	376.50±72.00	519.70±85.20	1512.90±83.90	750.20±57.10

	**0.8-5 kDa-D**	743.40±107.60	1208.30±49.60	628.30±87.00	653.60±106.30
	**<0.8 kDa-D**	664.90±139.70	726.80±69.60	886.30±218.80	894.20±16.70
	**D100%**	121.27±28.83	239.73±8.19	752.61±58.26	438.03±20.14
**1-pentanol**	**unfractionated-T**	5.00±2.60	6.00±0.80	13.50±0.50	87.60±6.00
	**>10 kDa-T**	1.30±0.70	2.30±0.40	5.50±1.40	19.60±0.70
	**5-10 kDa-T**	0.10±0.10	0.70±0.10	2.00±0.80	7.70±1.30
	**0.8-5 kDa-T**	3.10±0.20	3.90±0.60	8.20±1.90	40.90±4.30
	**<0.8 kDa-T**	3.90±0.60	4.80±0.70	8.10±0.40	21.70±2.10
	**T100%**	4.30±0.10	3.80±0.50	7.70±1.80	18.50±1.10
	**unfractionated-D**	27.20±14.10	91.10±10.10	158.90±6.50	136.30±8.30
	**>10 kDa-D**	14.20±2.00	18.60±2.50	62.40±7.40	440.40±22.60
	**5-10 kDa-D**	18.50±3.10	25.80±3.10	45.80±3.30	307.10±34.40
	**0.8-5 kDa-D**	40.90±2.70	76.10±3.90	163.90±6.70	407.10±88.10
	**<0.8 kDa-D**	32.70±5.00	36.40±3.20	104.70±20.20	313.20±13.90
	**D100%**	16.46±2.07	31.14±2.14	76.45±8.11	198.26±4.77
**2-hexenal**	**unfractionated-T**	3.40±0.40	4.50±0.60	6.80±0.20	37.80±4.20
	**>10 kDa-T**	3.60±1.00	3.50±0.20	4.80±1.00	10.20±2.10
	**5-10 kDa-T**	2.60±0.80	2.60±0.10	2.70±0.10	4.20±0.50
	**0.8-5 kDa-T**	3.00±0.10	3.20±0.30	5.50±2.00	18.30±3.10
	**<0.8 kDa-T**	2.00	2.50±0.20	4.00±1.40	6.40±0.70
	**T100%**	1.70	1.90±0.10	3.20±0.90	5.00±0.40
	**unfractionated-D**	82.00±6.90	93.50±18.80	47.30±2.10	48.00±5.50
	**>10 kDa-D**	10.40±1.00	29.60±1.00	115.60±45.30	162.80±7.60
	**5-10 kDa-D**	40.70±14.40	51.70±4.20	67.90±7.10	117.10±6.40
	**0.8-5 kDa-D**	108.60±32.00	86.60±10.80	66.20±13.30	142.20±9.80
	**<0.8 kDa-D**	77.70±5.80	55.30±14.20	39.80±2.80	83.00±5.40
	**D100%**	27.48±10.85	19.88±1.35	63.65±18.98	39.62±5.130
**Heptenal**	**unfractionated-T**	0.00	0.00	0.00	164.80±18.90
	**>10 kDa-T**	0.00	0.00	0.00	0.00
	**5-10 kDa-T**	0.00	0.00	0.00	0.00
	**0.8-5 kDa-T**	0.00	0.00	0.00	45.30±6.80
	**<0.8 kDa-T**	0.00	0.00	0.00	0.00
	**T100%**	0.00	0.00	0.00	1.40±2.50
	**unfractionated-D**	123.70±28.40	278.80±20.70	574.20±12.60	530.50±65.0
	**>10 kDa-D**	0.00	3.30±5.70	129.60±4.50	1323.90±52.60
	**5-10 kDa-D**	27.70±5.90	36.60±8.60	99.20±2.10	780.10±37.00
	**0.8-5 kDa-D**	146.90±17.60	237.00±0.60	504.60±9.40	1500.80±174.70
	**<0.8 kDa-D**	61.10±12.40	49.70±15.40	266.00±45.20	929.90±46.30
	**D100%**	2.08±3.61	7.44±2.17	123.36±12.75	401.10±12.36
**4-heptenal**	**unfractionated-T**	0.00	0.00	0.00	52.90±5.90
	**>10 kDa-T**	0.00	0.00	0.00	2.80±0.40
	**5-10 kDa-T**	0.00	0.00	0.00	0.00
	**0.8-5 kDa-T**	0.00	0.00	0.00	19.00±1.10
	**<0.8 kDa-T**	0.00	0.00	0.00	0.60±0.40
	**T100%**	0.00	0.00	0.00	0.00
	**unfractionated-D**	6.30±4.80	41.90±2.70	61.50±0.50	86.9±10.00
	**>10 kDa-D**	0.00	0.00	21.60±1.80	152.30±6.30
	**5-10 kDa-D**	0.00	4.50±1.40	15.70±1.30	133.50±7.30
	**0.8-5 kDa-D**	12.60±2.00	35.60±4.10	67.90±0.80	208.30±30.80
	**<0.8 kDa-D**	5.80±1.40	11.80±1.00	47.30±6.20	150.40±7.90
	**D100%**	0.00	1.36±0.26	27.33±1.68	86.81±1.88

2-heptenal	**unfractionated-T**	0.00	0.00	0.00	4.30±2.30
	**>10 kDa-T**	0.00	0.00	0.00	0.00
	**5-10 kDa-T**	0.00	0.00	0.00	0.00
	**0.8-5 kDa-T**	0.00	0.00	0.00	0.00
	**<0.8 kDa-T**	0.00	0.00	0.00	0.00
	**T100%**	0.00	0.00	0.00	0.00
	**unfractionated-D**	12.60±6.30	26.10±3.60	25.00±0.60	18.70±2.40
	**>10 kDa-D**	0.20±0.20	0.20±0.20	13.30±3.50	126.60±6.70
	**5-10 kDa-D**	2.60±0.70	4.60±0.70	11.70±0.20	71.10±3.20
	**0.8-5 kDa-D**	13.90±1.20	28.30±0.60	26.70±2.40	94.60±16.10
	**<0.8 kDa-D**	7.90±1.00	4.10±0.60	7.20±3.10	40.00±2.80
	**D100%**	7.40±1.41	7.26±0.57	19.00±1.35	37.17±0.72
1-octen-3-ol	**unfractionated-T**	0.00	0.00	0.00	128.30±13.30
	**>10 kDa-T**	0.00	0.00	0.00	0.00
	**5-10 kDa-T**	0.00	0.00	0.00	0.00
	**0.8-5 kDa-T**	0.00	0.00	0.00	44.50±3.50
	**<0.8 kDa-T**	0.00	0.00	0.00	0.00
	**T100%**	0.00	0.00	0.00	0.00
	**unfractionated-D**	25.60±4.60	164.20±9.70	196.00±3.00	169.80±17.10
	**>10 kDa-D**	0.00	5.30±1.60	84.80±13.90	500.80±17.00
	**5-10 kDa-D**	0.00	13.30±1.10	47.60±0.50	395.10±24.30
	**0.8-5 kDa-D**	21.30±4.40	113.40±2.30	187.90±12.70	454.90±74.90
	**<0.8 kDa-D**	9.80±1.30	31.90±3.90	100.10±24.0	258.50±12.30
	**D100%**	0.00	12.53±0.39	78.60±5.49	182.28±6.77
Octanal	**unfractionated-T**	0.00	0.00	0.00	88.50±10.30
	**>10 kDa-T**	0.00	0.00	0.00	7.90±3.10
	**5-10 kDa-T**	0.00	0.00	0.00	0.00
	**0.8-5 kDa-T**	0.00	0.00	0.00	35.90±5.60
	**<0.8 kDa-T**	0.00	0.00	0.00	10.20±1.20
	**T100%**	0.00	0.00	0.00	8.00±3.80
	**unfractionated-D**	122.40±42.20	198.40±18.50	369.00±6.30	322.40±34.40
	**>10 kDa-D**	32.70±8.80	38.10±5.20	106.50±12.80	734.80±40.70
	**5-10 kDa-D**	74.20±1.90	73.50±6.10	111.00±5.60	445.10±17.30
	**0.8-5 kDa-D**	143.70±9.60	175.70±4.60	305.90±1.30	906.20±108.90
	**<0.8 kDa-D**	88.40±10.90	67.20±9.30	195.80±32.70	552.50±36.20
	**D100%**	49.75±12.53	65.18±3.27	148.46±11.24	318.56±9.90
2,4 heptadienal	**unfractionated-T**	0.00	0.00	85.80±7.10	860.40±88.00
	**>10 kDa-T**	17.40±7.30	1.00±1.50	48.70±15.00	276.50±38.80
	**5-10 kDa-T**	0.00	0.00	0.00	76.60±34.10
	**0.8-5 kDa-T**	0.00	0.00	24.80±5.60	458.10±41.90
	**<0.8 kDa-T**	0.00	0.00	0.00	21.00±1.10
	**T100%**	0.00	0.00	61.10±31.30	259.70±37.10
	**unfractionated-D**	592.40±144.30	1039.70±225.70	1202.80±24.70	1653.10±77.80
	**>10 kDa-D**	123.90±25.70	225.30±12.70	920.40±383.90	3086.40±132.00
	**5-10 kDa-D**	200.20±21.70	403.40±33.30	987.90±45.70	2977.40±120.80
	**0.8-5 kDa-D**	538.80±31.80	1569.30±30.20	1685.30±281.60	3089.90±335.60
	**<0.8 kDa-D**	424.50±58.20	464.40±64.30	716.50±159.60	2510.70±101.80
	**D100%**	148.47±52.30	600.12±39.31	1714.96±7.07	2562.78±106.32
	**unfractionated-T**	0.60±0.30	3.10±0.90	15.40±1.80	44.80±18.10
	**>10 kDa-T**	1.20±0.50	2.60±1.00	6.00±1.70	14.70±3.30
	**5-10 kDa-T**	0.50±0.10	2.60±0.70	2.00±1.00	5.50±2.10

	**0.8-5 kDa-T**	1.00±0.40	2.80±0.20	5.20±2.40	28.80±5.70
**2,4 decadienal**	**<0.8 kDa-T**	0.30±0.10	0.60±0.20	1.40±0.10	5.30±0.40
	**T100%**	0.30±0.20	1.10±0.60	2.50±1.20	5.80±1.10
	**unfractionated-D**	17.40±0.70	88.50±28.30	225.80±7.90	306.10±15.20
	**>10 kDa-D**	7.80	28.60±2.50	4.50±2.80	282.70±65.30
	**5-10 kDa-D**	5.80±0.30	25.40±4.20	68.90±6.10	257.60±38.70
	**0.8-5 kDa-D**	19.20±1.50	136.20±2.10	220.00±67.20	607.50±121.20
	**<0.8 kDa-D**	7.70±2.40	22.10±4.60	125.30±52.10	381.90±37.30
	**D100%**	3.28±2.84	40.45±0.62	121.63±21.93	360.18±39.72

[1]Mean±standard deviation (n=3).

[*]D and T demonstrate the DATEM and Tween 20 respectively and indicate the surfactant and molecular weights show the corresponding fraction of potato protein hydrolysate used in the emulsion.

[A-G] Letters indicate significant differences between samples within the same day for 2-ethyl furan, 2-butenal, 1-penten-3-ol and hexanal (ANOVA, Tukey's post-hoc test, p<0.05).

[a-d] Letters indicate significant changes during storage for 2-ethyl furan, 2-butenal, 1-penten-3-ol and hexanal (ANOVA, Tukey's post-hoc test, p<0.05).
